# Longitudinal cytokine and multi-modal health data of an extremely severe ME/CFS patient with HSD reveals insights into immunopathology, and disease severity

**DOI:** 10.3389/fimmu.2024.1369295

**Published:** 2024-04-08

**Authors:** Fereshteh Jahanbani, Justin Cyril Sing, Rajan Douglas Maynard, Shaghayegh Jahanbani, Janet Dafoe, Whitney Dafoe, Nathan Jones, Kelvin J. Wallace, Azuravesta Rastan, Holden T. Maecker, Hannes L. Röst, Michael P. Snyder, Ronald W. Davis

**Affiliations:** ^1^ Department of Genetics, Stanford University School of Medicine, Stanford, CA, United States; ^2^ Department of Molecular Genetics, Donnelly Center, University of Toronto, Toronto, ON, Canada; ^3^ Division of Immunology and Rheumatology, Stanford University School of Medicine, Veterans Affairs (VA) Palo Alto Health Care System, Palo Alto, CA, United States; ^4^ ME/CFS Collaborative Research Center at Stanford, Stanford Genome Technology Center, Stanford University School of Medicine, Palo Alto, CA, United States; ^5^ Sean N. Parker Center for Allergy and Asthma Research at Stanford University, Pulmonary and Critical Care Medicine, Institute of Immunity, Transplantation, and Infectious Diseases, Stanford University, Palo Alto, CA, United States

**Keywords:** ME/CFS, EDS/hEDS/HSD, POTS, MCAS, MCS, longitudinal omics, Th2-cytokines, complex chronic condition

## Abstract

**Introduction:**

Myalgic Encephalomyelitis/Chronic Fatigue Syndrome (ME/CFS) presents substantial challenges in patient care due to its intricate multisystem nature, comorbidities, and global prevalence. The heterogeneity among patient populations, coupled with the absence of FDA-approved diagnostics and therapeutics, further complicates research into disease etiology and patient managment. Integrating longitudinal multi-omics data with clinical, health,textual, pharmaceutical, and nutraceutical data offers a promising avenue to address these complexities, aiding in the identification of underlying causes and providing insights into effective therapeutics and diagnostic strategies.

**Methods:**

This study focused on an exceptionally severe ME/CFS patient with hypermobility spectrum disorder (HSD) during a period of marginal symptom improvements. Longitudinal cytokine profiling was conducted alongside the collection of extensive multi-modal health data to explore the dynamic nature of symptoms, severity, triggers, and modifying factors. Additionally, an updated severity assessment platform and two applications, ME-CFSTrackerApp and LexiTime, were introduced to facilitate real-time symptom tracking and enhance patient-physician/researcher communication, and evaluate response to medical intervention.

**Results:**

Longitudinal cytokine profiling revealed the significance of Th2-type cytokines and highlighted synergistic activities between mast cells and eosinophils, skewing Th1 toward Th2 immune responses in ME/CFS pathogenesis, particularly in cognitive impairment and sensorial intolerance. This suggests a potentially shared underlying mechanism with major ME/CFS comorbidities such as HSD, Mast cell activation syndrome, postural orthostatic tachycardia syndrome (POTS), and small fiber neuropathy. Additionally, the data identified potential roles of BCL6 and TP53 pathways in ME/CFS etiology and emphasized the importance of investigating adverse reactions to medication and supplements and drug interactions in ME/CFS severity and progression.

**Discussion:**

Our study advocates for the integration of longitudinal multi-omics with multi-modal health data and artificial intelligence (AI) techniques to better understand ME/CFS and its major comorbidities. These findings highlight the significance of dysregulated Th2-type cytokines in patient stratification and precision medicine strategies. Additionally, our results suggest exploring the use of low-dose drugs with partial agonist activity as a potential avenue for ME/CFS treatment. This comprehensive approach emphasizes the importance of adopting a patient-centered care approach to improve ME/CFS healthcare management, disease severity assessment, and personalized medicine. Overall, these findings contribute to our understanding of ME/CFS and offer avenues for future research and clinical practice.

## 1 Introduction

Myalgic encephalomyelitis/chronic fatigue syndrome (ME/CFS) is a chronic, complex, and debilitating multi-system disease affecting millions of people worldwide (1–3). ME/CFS is characterized by post-exertional malaise, manifested as the worsening of existing symptoms or the onset of new ones following mental or physical exertion, and exposure to environmental risk factors. Persistent symptoms include, but are not limited to, cognitive impairments, hypersensitivity to various stimuli (4–7), headaches, muscle and joint pain, sore throat, tender lymph nodes, gastrointestinal issues, chills, night sweats, multi-chemical sensitivity, shortness of breath, irregular heartbeat, sleep disturbance, pain, and orthostatic intolerance (8–10). Symptoms tend to manifest in various combinations and levels of intensity for each patient, often marked by periods of remission and relapse. This variability, along with the gradual worsening of symptoms can lead to a substantial reduction in patients’ ability to engage in previous levels of occupational, educational, social, and personal activities, and quality of life. Around 25% of ME/CFS patients, including children, may become severely affected (11), confined to their homes or beds for extended periods, with some becoming critically ill (12–20).

Critically ill ME/CFS patients may require life-supporting devices like jejunal feeding tubes or central venous catheters (19) for nutrient delivery. Despite their importance, these devices carry significant health risks, contributing to morbidity and mortality, including thrombosis, infections, intestinal perforation, and metabolic complications (21–23), thereby further complicating the management of the patients’ health.

Despite decades of research, the precise pathophysiology of ME/CFS remains unclear. The limited understanding of its underlying causes, the FDA-approved diagnostic tests, and treatments, have presented significant challenges in effectively diagnosing and treating ME/CFS patients (9). These substantial hurdles underscore the necessity for innovative research approaches to address the existing gap in disease etiology and enhance patient care and quality of life (24).

A substantial body of research has implicated genetic variations and environmental stressors, such as infections, trauma, and exposure to toxins, in the etiology of ME/CFS (25–30). Additionally, ME/CFS patients and their families exhibit a higher prevalence of conditions like Ehlers-Danlos syndrome (EDS), hypermobility spectrum disorders (HSD) (31), post-treatment Lyme disease (PTLDS) (32), and Pediatric Acute-onset Neuropsychiatric Syndrome (PANS) (33–35). Recent investigations into post-COVID syndrome have uncovered significant symptom overlap between ME/CFS and Long COVID (36–38). Studying shared biological systems across these comorbidities holds promise for biomarker and therapeutic discoveries (39).

Within the intricate tapestry of ME/CFS and its comorbidities, a central point of agreement in the scientific community revolves around immune dysregulation (40) and aberrant cytokine expression. However, a thorough examination of the literature reveals inconsistencies, with some studies indicating an overactive immune response leading to chronic inflammation (41–43), while others propose a scenario of immune system debilitation (44).

The inconsistency in research findings could be due to a myriad of factors including the dynamic nature of ME/CFS (10), characterized by relapse and remission cycles, diverse triggers, and variable durations, as well as medication, lifestyle, and comorbidities (31, 35, 45–48). Adding to the complexity is the subjective nature of symptomatology, spanning across multiple bodily systems, and the inherent limitations in tools available for the quantitative measurement of severity fluctuations.

Currently, to assess clinical severity, researchers rely on methods such as the SF-36 questionnaire, and cardiopulmonary exercise tests, categorizing patients into mild, moderate, and severe groups. Acknowledging the spectrum’s breadth, a recent addition of a “very severe” category attempts to capture the extreme end of the scale (5, 20). However, as the condition worsens, some patients transition from ambulatory to bed bound states, rendering conventional assessment tools impractical (49, 50) and highlighting the pressing need for more adaptable approaches. The exclusion of the very severely ill cohort from mainstream ME/CFS studies can also result in misclassification, contributing to research inconsistency and impeding our understanding of disease pathophysiology (49, 50). Recognizing this critical gap, we initiated a comprehensive longitudinal study that integrates clinical, health, pharmaceutical, nutraceutical, textual, and cytokine data, and harnesses the power of artificial intelligence (AI). Our focus on an extremely severe ME/CFS patient in a phase of marginal improvement has provided a unique perspective on the underlying immunopathology and its connections to symptomatology, severity, and comorbidity in ME/CFS. By delving into the intricacies of severity and incorporating the experiences of the most severely affected individuals, our objective is to lay the groundwork for substantial advancements in the quality of care for those navigating the challenges of ME/CFS. Myalgic encephalomyelitis/chronic fatigue syndrome (ME/CFS) is a chronic, complex, and debilitating multi-system disease affecting millions of people worldwide (1–3). ME/CFS is characterized by post-exertional malaise, manifested as the worsening of existing symptoms or the onset of new ones following mental or physical exertion, and exposure to environmental risk factors. Persistent symptoms include, but are not limited to, cognitive impairments, hypersensitivity to various stimuli (4–7), headaches, muscle and joint pain, sore throat, tender lymph nodes, gastrointestinal issues, chills, night sweats, multi-chemical sensitivity, shortness of breath, irregular heartbeat, sleep disturbance, pain, and orthostatic intolerance (8–10). Symptoms tend to manifest in various combinations and levels of intensity for each patient, often marked by periods of remission and relapse. This variability, along with the gradual worsening of symptoms can lead to a substantial reduction in patients’ ability to engage in previous levels of occupational, educational, social, and personal activities, and quality of life. Around 25% of ME/CFS patients, including children, may become severely affected (11), confined to their homes or beds for extended periods, with some becoming critically ill (12–20).

Critically ill ME/CFS patients may require life-supporting devices like jejunal feeding tubes or central venous catheters (19) for nutrient delivery. Despite their importance, these devices carry significant health risks, contributing to morbidity and mortality, including thrombosis, infections, intestinal perforation, and metabolic complications (21–23), thereby further complicating the management of the patients’ health.

Despite decades of research, the precise pathophysiology of ME/CFS remains unclear. The limited understanding of its underlying causes, the FDA-approved diagnostic tests and treatments, have presented significant challenges in effectively diagnosing and treating ME/CFS patients (9). These substantial hurdles underscore the necessity for innovative research approaches to address the existing gap in disease etiology and enhance patient care and quality of life (24).

A substantial body of research has implicated genetic variations and environmental stressors, such as infections, trauma, and exposure to toxins, in the etiology of ME/CFS (25–30). Additionally, ME/CFS patients and their families exhibit a higher prevalence of conditions like Ehlers-Danlos syndrome (EDS), hypermobility spectrum disorders (HSD) (31), post-treatment Lyme disease (PTLDS) (32), and Pediatric Acute-onset Neuropsychiatric Syndrome (PANS) (33–35). Recent investigations into post-COVID syndrome have uncovered significant symptom overlap between ME/CFS and Long COVID (36–38). Studying shared biological systems across these comorbidities holds promise for biomarker and therapeutic discoveries (39).

Within the intricate tapestry of ME/CFS and its comorbidities, a central point of agreement in the scientific community revolves around immune dysregulation (40) and aberrant cytokine expression. However, a thorough examination of the literature reveals inconsistencies, with some studies indicating an overactive immune response leading to chronic inflammation (41–43), while others propose a scenario of immune system debilitation (44).

The inconsistency in research findings could be due to a myriad of factors including the dynamic nature of ME/CFS (10), characterized by relapse and remission cycles, diverse triggers, and variable durations, as well as medication, lifestyle, and comorbidities (31, 35, 45–48). Adding to the complexity is the subjective nature of symptomatology, spanning across multiple bodily systems, and the inherent limitations in tools available for the quantitative measurement of severity fluctuations.

Currently, to assess clinical severity, researchers rely on methods such as the SF-36 questionnaire, and cardiopulmonary exercise tests, categorizing patients into mild, moderate, and severe groups. Acknowledging the spectrum’s breadth, a recent addition of a “very severe” category attempts to capture the extreme end of the scale (5, 20). However, as the condition worsens, some patients transition from ambulatory to bed bound states, rendering conventional assessment tools impractical (49, 50) and highlighting the pressing need for more adaptable approaches. The exclusion of the very severely ill cohort from mainstream ME/CFS studies can also result in misclassification, contributing to research inconsistency and impeding our understanding of disease pathophysiology (49, 50). Recognizing this critical gap, we initiated a comprehensive longitudinal study that integrates clinical, health, pharmaceutical, nutraceutical, textual, and cytokine data, and harnesses the power of artificial intelligence (AI). Our focus on an extremely severe ME/CFS patient in a phase of marginal improvement has provided a unique perspective on the underlying immunopathology and its connections to symptomatology, severity, and comorbidity in ME/CFS. By delving into the intricacies of severity and incorporating the experiences of the most severely affected individuals, our objective is to lay the groundwork for substantial advancements in the quality of care for those navigating the challenges of ME/CFS.

## 2 Materials and methods

### 2.1 Study design and participant

**Figure 1:**
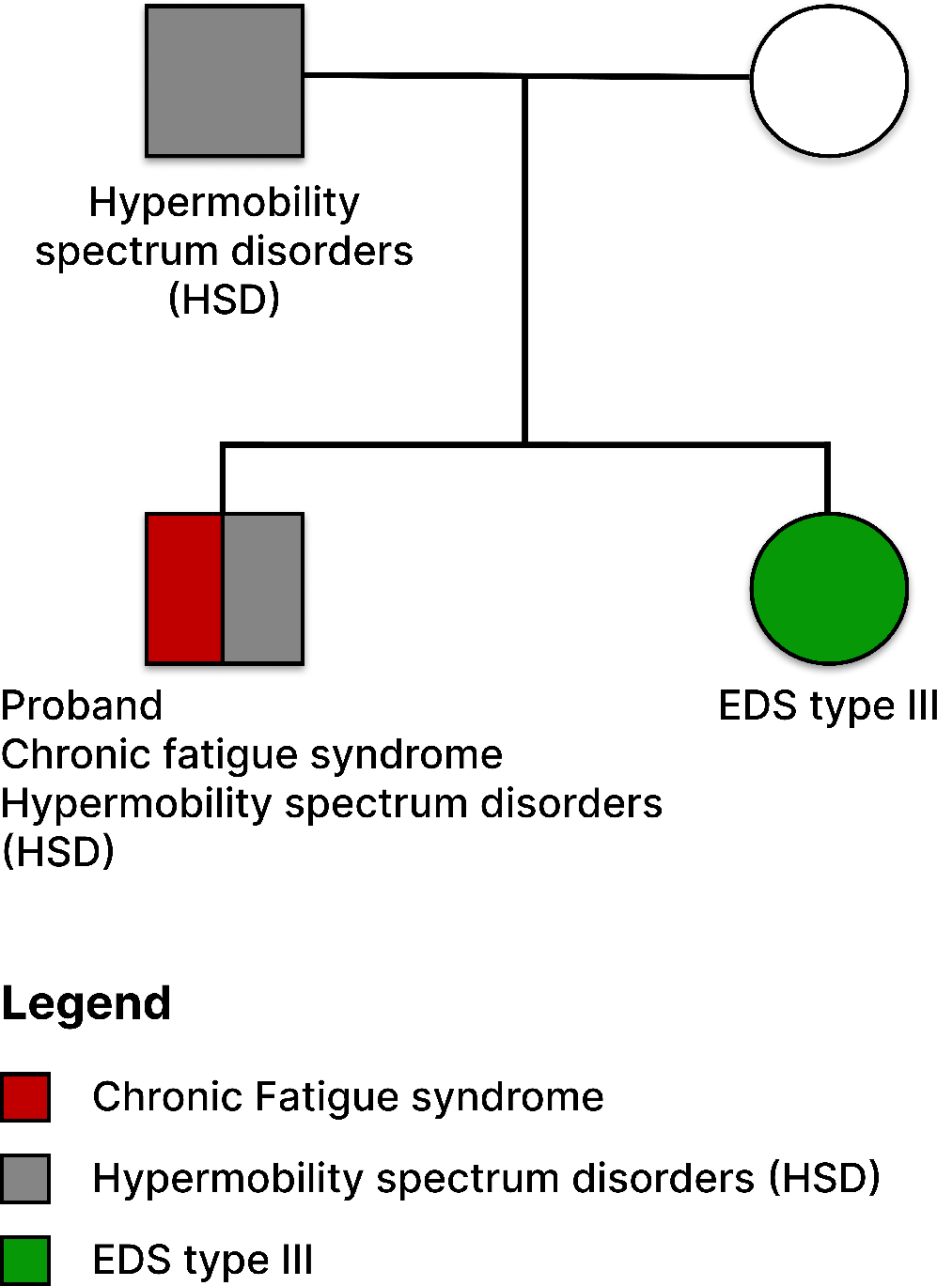
Pedigree Structure of the Family with ME/CFS, EDS Type III, and HSD History. The depicted pedigree illustrates an ME/CFS patient of Caucasian descent. The patient presents with the comorbidity of Hypermobility Spectrum Disorder (HSD) and shares a family history of Ehlers-Danlos Syndrome (EDS) Type III. The sister with confirmed EDS Type III is highlighted in green.

Over the course of four years, from June 2017 (when the participant was 34) to January 2021, we collected longitudinal blood samples from an extremely ill male ME/CFS patient. These samples were used for integrative case study on longitudinal plasma cytokine profiling, which incorporated health, clinical, and textual data to identify cytokines associated with disease severity. The patient began experiencing health issues at age 21 and received a diagnosis of ME/CFS almost a decade later. He is entirely bedridden, relying on caregivers for all aspects of his life, and receives nutrition via a G-tube and PICC line. The patient, of Caucasian descent, also has a comorbidity of hypermobility spectrum disorder, with a family history of EDS type III and HSD (refer to **Figure 1**). Throughout this four-year period, from June 2017 to January 2021, the patient’s extreme hypersensitivity and PEM improved to the extent that he could tolerate human presence and regain access to internet use and social media.

This patient met various diagnostic criteria outlined by renowned ME/CFS doctors, such as those from the International Consensus Criteria and Canadian Consensus Criteria. These criteria encompassed Post-Exertional Malaise (PEM), symptom duration (persisting for over six months), multiple symptoms (including gastrointestinal issues, POTS, muscular pain, cold intolerance, sleep impairment, and sensory intolerance), functional impairment (severely affecting daily life), and exclusion of alternative diagnoses (validated through medical investigations) (51).

### 2.2 Longitudinal plasma sample collection

**Figure 2:**
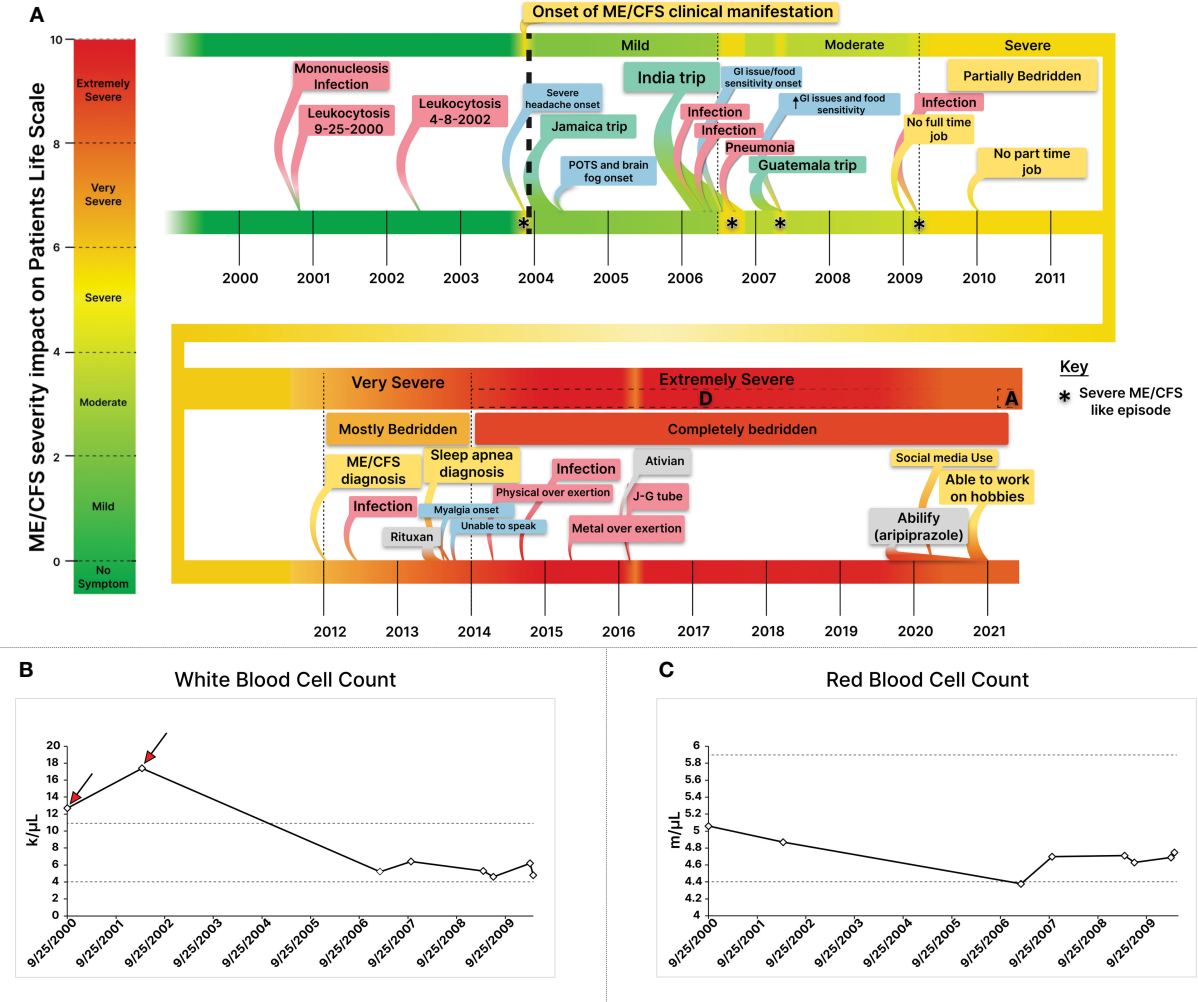
Constructing a Comprehensive Disease Timeline through the Integration of Longitudinal Health and Clinical Data for An Extremely Severe ME/CFS Patient. **(A)** Integrating health and clinical data illustrates dynamic severity changes, triggers, contributing factors, and new symptom onsets. Infections like mononucleosis and stressors such as infections, medications, and trauma modulate severity. **(B)** Retrospective analysis of Longitudinal Complete Blood Count (CBC) data from the extremely severe ME/CFS patient during the first decade of his illness revealed two episodes of leukocytosis (red arrows) prior to the onset of health symptom, suggesting infection-derived immune dysregulation as a potential trigger for his ME/CFS. **(C)** Leukocytosis was followed by a lasting red blood cell count reduction. While white blood cell counts normalized, RBC levels declined, remaining low. * Depicts a Severe ME/CFS-like episode.

Over the course of four years (2017-2021), a total of nine plasma samples were collected from an extremely severe ME/CFS patient, transitioning to a marginal improvement in health (**Figure 2A**; **Table 1**). Blood samples were obtained using BD-K2EDTA Vacutainer purple top 10 mL tubes (366643) and were immediately mixed by gently and thoroughly inverting the tube five to ten times. Subsequently, plasma was separated through centrifugation at 2500 RPM for 10 minutes at 4°C. The isolated plasma layer was transferred into 15 ml polypropylene conical tubes, thoroughly mixed, and then aliquoted into 1.5 ml DNase- and RNase-free Eppendorf tubes. These aliquoted plasma samples were promptly snap-frozen in liquid nitrogen and stored at −80°C until the time of assay.

**Table 1:** Longitudinal collection of plasma cytokines, age, and severity score over the 4-Year study period.

Date	Age	CFS severity
6/29/17	33	Severe D
6/18/18	34	Severe D
4/9/19	35	Severe D
8/1/19	35	Severe D
12/19/19	36	Severe D
1/14/20	36	Severe D
2/7/20	36	Severe D
2/13/20	36	Severe D
1/18/21	37	Severe A

Nine plasma samples were collected using EDTA tubes, capturing the nuanced progression of the patient’s health from the extremely severe stage D to A. The collected plasma samples underwent prompt processing and were stored at -80°C.

### 2.3 Longitudinal plasma cytokine profiling using multiplex cytokine bead array

The analysis was conducted in collaboration with the Stanford Human Immune Monitoring Center, employing the MILLIPLEX Human Cytokine/Chemokine Immunology Multiplex Assay. The assay utilized kits purchased from EMD Millipore Corporation, Burlington, MA, and followed the manufacturer’s recommendations with some modifications. The H80 kits consisted of three panels: Panel 1 (Milliplex HCYTA-60K-PX48), Panel 2 (Milliplex HCP2MAG-62K-PX23), and Panel 3 (Milliplex HSP1MAG-63K-06 and HADCYMAG-61K-03, targeting Resistin, Leptin, and HGF) to generate a 9-plex.

Following the recommended protocol, samples were diluted threefold for cytokines measured in Panel 1 & 2 and tenfold for those measured in Panel 3, based on the abundance range of each cytokine in plasma. A 25 µl volume of the diluted sample was mixed with antibody-linked magnetic beads in a 96-well plate and incubated overnight at 4°C with shaking. Incubation steps were performed on an orbital shaker at 500-600 rpm at both cold and room temperature. After washing the plates twice with a wash buffer using a Biotek ELx405 washer (BioTek Instruments, Winooski, VT), a one-hour incubation at room temperature with biotinylated detection antibody was conducted. Streptavidin-PE was then added for 30 minutes while shaking.

Plates were washed again, and PBS was added to wells for reading in the Luminex FlexMap3D Instrument with a lower bound of 50 beads per sample per cytokine. Control beads (Custom Assay Chex, Radix Biosolutions, Georgetown, Texas) were included in all wells. Wells with a bead count less than 50 were flagged, and data with a bead count less than 20 were excluded. Data analysis was performed using MasterPlex software (Hitachi Software Engineering America Ltd., MiraiBio Group), with both median fluorescence intensity (MFI) and calculated concentration values reported for each analyte.

### 2.4 Statistical analyses

All statistical analyses and visualization were performed in R using custom R scripts. The raw cytokine MFI (median fluorescence intensity) values were log 2 transformed and used for downstream analysis. Hierarchical clustering was done on the samples and cytokines using euclidean distance, and the ward.D2 clustering method. Outliers in cytokine profiling data were identified using the Z-score method, which provides a standardized measure of deviation from the mean, facilitating the detection of biologically meaningful differences. The robustness of the Z-score method to variations in sample size and distribution made it suitable for our study (52). Z-scores were calculated by comparing the latest time-point sample (most improved health state; 01/18/2021) cytokine levels to the average of all the previous time-points. P-values were generated from two-tailed normal distribution samples from each z-score, followed by FDR p-value adjustment for multiple-hypothesis testing. In addition, we conducted an analysis to investigate the correlation between cytokine intensity and the health state over time in the patient. The Pearson correlation coefficient was employed as a statistical measure to assess the strength and direction of the linear association between cytokine levels and health state. We performed a similar analysis to assess the correlation between the change in medication over time and health state.

### 2.5 Patient blog post sentiment analysis

We conducted a thorough analysis on blog posts authored by the extremely severe ME/CFS patient. The extracted dataset included essential details like publication dates and post content. Data preprocessing involved extracting and standardizing publication dates while accounting for time zone variations. The datasets were then filtered for the timeframe spanning 2017 to 2021, aligning precisely with the longitudinal plasma collection phase and coinciding with the period of marginal health improvement. Our exploratory data analysis focused on word frequency at monthly and yearly intervals. We also investigated post lengths over time, calculating and visualizing average post lengths. Employing a pre-trained large language transformer model (SamLowe/roberta-base-go_emotions, available from HuggingFace (53), we conducted sentiment analysis, and the outcomes were visually depicted through monthly and yearly sentiment trends.

To deepen our understanding of linguistic nuances, word cloud analysis was applied, generating visually intuitive representations of word frequencies in all posts, both on a monthly and yearly basis. Furthermore, topic modeling was applied using natural language processing and a latent dirichlet allocation model to uncover hidden thematic structures across the blog posts. The complete pipeline, encompassing data extraction to exploratory analyses, was executed using Python. Leveraging sentiment analysis and topic modeling, the outcomes are encapsulated in a user-friendly Streamlit-powered web application, LexiTime (Lexical and Temporal Insight Mining Exploration). The application is accessible at https://github.com/singjc/lexitime.

### 2.6 Development of a web-based application for real-time symptom tracking and intervention assessment

A web-based application was developed for the active tracking of symptoms for ME/CFS patients. The application was developed using a Node.js frontend and Python backend, interacting with a MongoDB and Postgres data system to save patient responses. The UI/UX design phase focused on creating an intuitive and visually appealing interface for seamless symptom tracking. User feedback and iterative design processes were implemented to enhance user experience, ensuring accessibility and ease of use.

Given the sensitive nature of health data,robust security measures have been implemented at multiple layers of the application to ensure data confidentiality, integrity, and availability. The security design adhered to established principles (54–57) and included end-to-end encryption, input sanitization, server-side authentication, and encryption of usernames and passwords. We employed HTTPS for transport in the web-based application and AES-256 for data encryptions. The application underwent rigorous testing phases, ensuring functionality and usability. User feedback and testing results informed iterative development cycles, ensuring a robust and reliable web-based tool. Upon successful testing, the web-based application was deployed to a secure server, ensuring accessibility for users. Cross-browser compatibility and responsive design were prioritized to facilitate usage across various devices.

## 3 Results

### 3.1 Constructing a comprehensive disease timeline through integration of longitudinal health and clinical data

**Figure 3:**
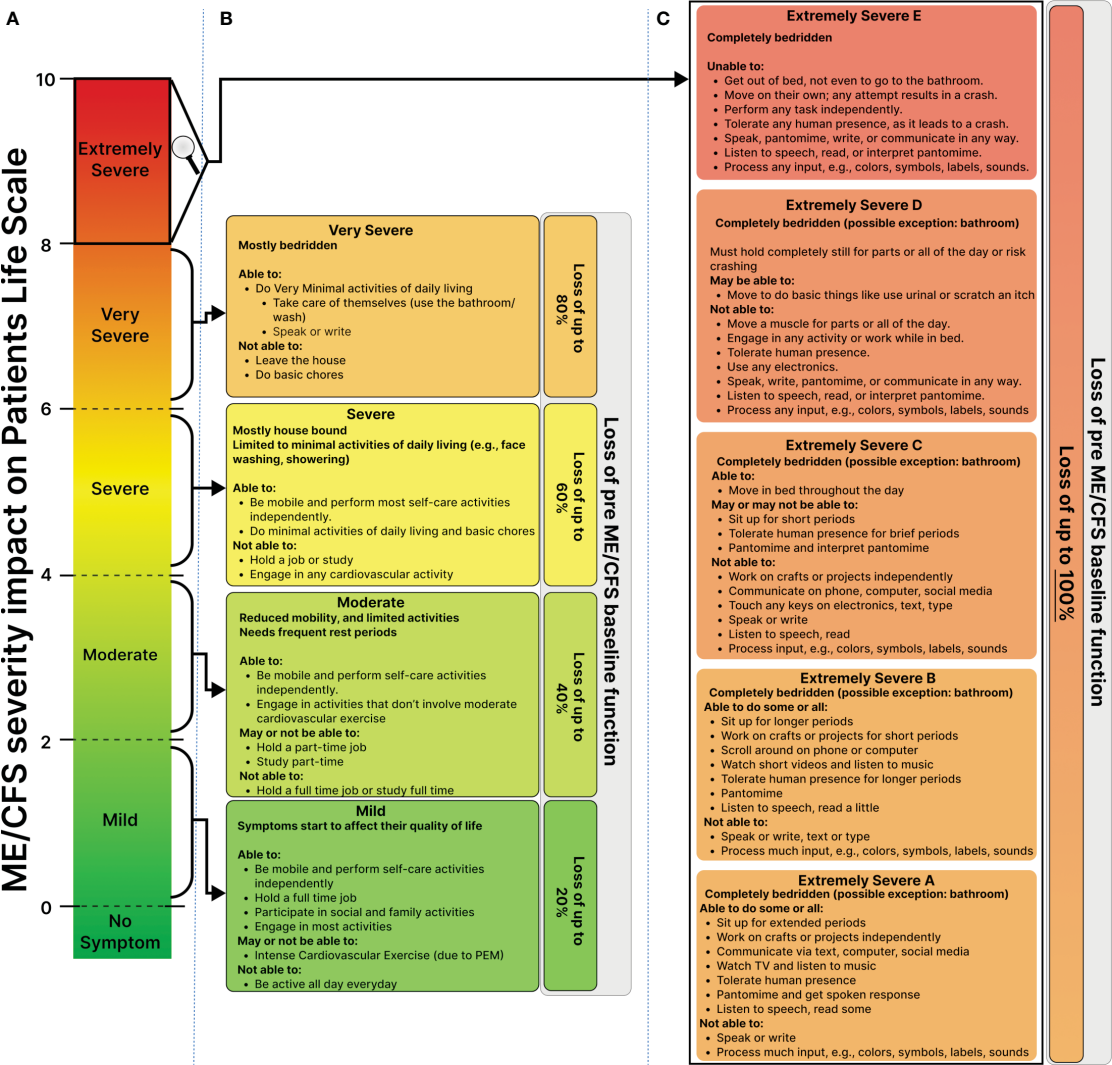
Proposed Framework for Personalized Severity Assessment in ME/CFS to Capture Variation in ME/CFS Severity and Life Impairment across Patients and Time. **(A)** Illustrates the dynamic range of the ME/CFS severity scale based on the disease’s impact on all aspects of the patient’s life, including occupational, educational, social, and personal spheres. **(B)** Depicts the impact of mild to severe ME/CFS on the patient’s life. Mild: maintained about 80% of pre-ME/CFS functional capacity, as well as full-time employment with limitations due to post-exertional malaise (PEM). Moderate: pre-ME/CFS functional capacity, unable to hold part-time work, with increased limitations in activity, progressing to severe: inability to hold any job, primarily house and bedbound. **(C)** Shows the patient’s functioning ability significantly degrading from extremely severe A to D, highlighting ME/CFS’s profound impact at this level. Severe nutritional deficiencies led to Gastrostomy tube (G-tube) and Peripherally inserted central catheter (PICC Line) Line use. Sensory intolerance intensified, making it impossible for the patient to tolerate others in his room. At stage D, communication loss and internet access loss intensified social isolation.

Individuals with very severe ME/CFS encounter communication challenges, impeding health evaluation and therapeutic assessment. We took a collaborative approach with patients’ caregivers involving meticulous utilization of patient and caregiver notes, alongside clinical health records, to construct a detailed disease timeline and severity assessments (**Figure 2**, **Table 1**, **Figure 3**).

The patient began experiencing health symptoms in 2004 and was diagnosed with ME/CFS in 2012 at the age of 29, after struggling with health issues for nearly a decade (**Figure 2A**). He had an active life prior to his illness. Longitudinal clinical blood cell count (CBC) analysis indicated two episodes of leukocytosis, characterized by an elevated white blood cell (WBC) count in 2000 and 2002 (**Figure 2B**), which occurred before the onset of experiencing health symptoms. The initial leukocytosis was attributed to EBV-mediated mononucleosis, as confirmed by a monospot test, and may have potentially acted as a trigger for the condition (**Figure 2A**). Longitudinal analyses of clinical data revealed mononucleosis as the cause for his initial leukocytosis. Retrospective complete blood count (CBC) analyses also illustrated a consistently diminished red blood cell count subsequent to the leukocytosis, persisting at a lower or below-normal range throughout the course of the illness (**Figure 2B**).

In 2006, during a trip to India, the patient developed a peculiar cold with mild symptoms lasting two weeks. Four months later, he encountered an abrupt onset of symptoms akin to severe ME/CFS cases (**Figure 2A**). After a slow recovery, regaining about 60% of health (patient self-assessment) (**Figure 2A**), his disease severity transitioned from mild to moderate (**Figure 2A**). Subsequently, a similar episode unfolded, starting with mild diarrhea, and escalating to severe ME/CFS, rendering him bedridden, only able to consume liquified white rice soup. Three months later, he ended up with pneumonia in a hospital in India, forcing him to return home. Upon return, although he partially regained health, lingering symptoms including lightheadedness persisted. Seeking a remedy, he traveled to Guatemala, where another severe ME/CFS-like episode occurred, mirroring the India experience. It began with mild diarrhea after a meal, followed by what the patient self-described as a stomach shutdown, possibly indicative of gastroparesis, and fatigue. After a week in this condition, he returned home, and his condition improved. However, his health baseline never fully recovered (patient self-assessment), and issues like gastroparesis, postprandial nausea, cold intolerance, and cold extremities surfaced (**Figure 2A**).

Throughout the first decade of his ME/CFS journey, the patient’s symptom severity gradually worsened from mild to moderate (2004-2006: mild, 2006-2010: moderate) (**Figure 2A**), with a notable presence of postural orthostatic intolerance (POTS), which significantly worsened after cardiovascular exercise and reappeared during post-remission periods (**Figure 2A**). In 2009, while managing only a part time job, the patient encountered another cold episode with initially mild symptoms. This episode triggered a decline in his health, reminiscent of the severe ME/CFS-like states he had previously experienced (**Figure 2A**). While mostly housebound, he managed short walks for groceries, performed self-care activities, cooked, and was sufficient. His condition further advanced from moderate to severe, rendering him bed bound. Describing his main symptoms as a ‘total body shut down,’ he emphasized the severity of the debilitating post-exertional malaise (**Figure 2A**). To articulate the gravity of his condition, he offered the analogy: “To compare the state I was in in 2012 to staying up for two nights in a row while fasting, then getting drunk. The state you would be in on the third day — hangover, not having slept or eaten in 3 days — is close, but still better than many ME/CFS patients feel every day”.

In 2013, the patient received Rituximab, after which he experienced an extreme crash and became permanently affected by PEM (**Figure 2A**). Muscular pain developed in the back of his legs, hindering his ability to stand or walk short distances (**Figure 2A**). Subsequently, he lost the capacity to speak, and communicated by text messages and a pre-programmed app for basic communication and food requests, ultimately transitioning to a routine food delivery program to minimize the need for texting (18).

During this period, he exerted minimal activity including lying on a lawn chair and listening to music with headphones for a few hours before returning to his room, with 2–3 short trips to the kitchen comprising the extent of his daily walking. Later, he obtained a wheelchair to facilitate movement to the kitchen (**Figures 2A**, **3A, B**). However, a physically demanding event in 2014, coupled with emotional strain, rendered him fully bedridden and intolerant of human contact, escalating his condition to an extremely severe state overnight (**Figures 2A**, **3C**). Transitioning from a very severe to an extremely severe state, heightened sensorial intolerance to noise, human contact, and social media impeded the patient’s ability to receive basic necessities. To cope, he resorted to covering his eyes with a folded trowel and wearing earphones and earmuffs to block outside stimuli (18) (**Figure 3**). The patient coined the term “mental crash” to describe this sensorial intolerance, emphasizing that even stimuli unrelated to any mental links could overwhelm the brain’s processing capacity. He subsequently isolated himself from human presence and the external world (**Figure 3C**) (18).

During emergencies intravenous Ativan administration enabled basic communication (**Figure 2A**). In the fall of 2019, the patient began a low dose of Abilify (aripiprazole, 0.2 mg), which was gradually increased to 2 mg by February 17, 2020. By June 2020, he reported slight improvement, allowing him to communicate briefly in writing using his cell phone. In one of his posts, the patient attributed this improvement to several factors, including an increase in the doses of Cortef (hydrocortisone) from 10 to 15 (to address potential adrenal insufficiency), and the Abilify treatment. In late 2020, he reported being able to have people in his room without the need for earphones and earmuffs (18) (**Figures 2A**, **3C**).

### 3.2 A proposed framework for assessing the personalized severity of ME/CFS

We built upon the existing classifications of mild to very severe (9), but also introduced the “extremely severe” category, further subdividing it into subclasses (A, B, C, D, and E) (**Figure 3**). The resulting framework classifies patients on a 1-10 scale and is defined around the impact of symptoms on patients’ ability to regain their pre-ME/CFS baseline function at a single symptom resolution.

The mild stage is defined in which the patient maintains 80% of their baseline function and full-time employment, albeit with limitations due to post-exertional malaise. Progressing to a moderate stage reduces their capacity to function to 60% or lower of their baseline, limiting them to part-time work.

Transitioning to the severe stage renders patients house and bed-bound, unable to work. They can perform most self-care activities independently but are unable to hold a job or engage in cardiovascular activity. At a very severe stage, patients become mostly bedridden, only able to perform minimal activities of daily living and self-care, such as bathing. They are unable to leave the house or do basic chores.

Extremely severe ME/CFS significantly interferes with nearly all aspects of the patient’s life (**Figure 3C**). Sensory intolerance and gastrointestinal disorders can cause severe nutritional deficiencies, resulting in a significant weight drop and the potential use of a G-J tube and PICC line for nutritional and medicinal support. From stage B to D, sensory intolerance makes it impossible for the patient to tolerate others in their room. At stage D, they lose communication abilities and internet access, intensifying social isolation.

### 3.3 Development of ME-CFSTrackerApp, a web-based application for real-time symptom tracking and intervention assessment

As a companion to our work, a web-based application was developed to allow patients to create an electronic journal of their symptoms, medications, and life events. The application was built using a stack of a Node.js front end and a Python back end and deployed on a secure server hosted at Stanford. The deployment is configured to guarantee constant uptime and cross-browser compatibility to ensure accessibility. Cross-browser compatibility and responsive design were prioritized to facilitate seamless usage across various devices, enhancing the accessibility and user experience. To promote widespread usage and accessibility, ME-CFSTrackerApp is freely available for use. Users can navigate to the application using the provided link “https://me-cfstrackerapp.su.domains”, where they can benefit from its features without any cost. This commitment to open access ensures that individuals can readily leverage the application for their real-time symptom tracking and intervention assessment needs.

### 3.4 Longitudinal cytokine analyses

**Figure 4:**
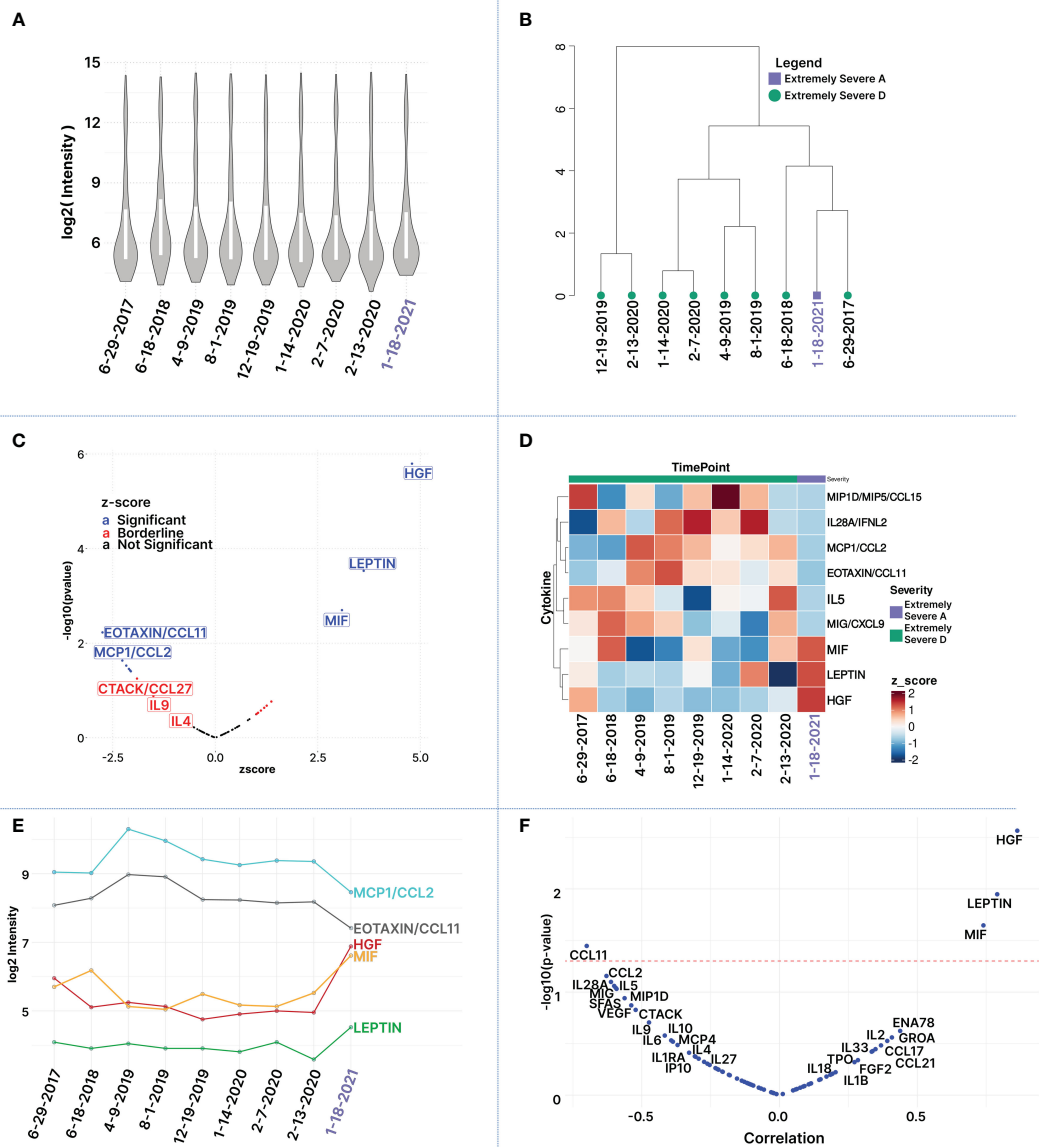
Longitudinal Multiplex Cytokine Profiling. **(A)** Distribution of log2-transformed cytokine MFI values per sample: Each boxplot represents a time point (run in 3 cytokine panels). The boxplot is arranged chronologically. **(B)** Hierarchical Clustering of Samples: This panel reveals groupings of similar time points and illustrates relationships between the samples based on clustering of log2-transformed cytokine intensities. **(C)** Volcano Plot of Differentially Expressed Cytokines: Plot illustrates cytokines based on the z-score derived from the last time point (Jan. 21), corresponding to the patient’s improved severity to ‘extremely severe A,’ in comparison to the average of the preceding nine time points. The x-axis represents z-scores, while the y-axis line represents -log (p-value). Black and blue dots marked cytokines with z-score values within -1 and 1, and p-value<0.05, respectively. **(D)** Heatmap of Top Differentially Expressed Cytokines: Columns (sample time points) and rows (cytokines) are clustered using euclidean distance and ward.D2 clustering. Most cytokines are reduced in the healthiest time point (marked in purple) except MIF, HGF, and LEP. Dark blue signifies the lowest z-scores, dark red the highest. **(E)** Log2 intensity levels of top 5 differentially expressed cytokines over time demonstrates cytokines that share similar trends. **(F)** Pearson correlation of cytokines in relation to the health state of the patient at the 9 different timepoints. The red dashed line indicates a 5% p-value cutoff.

For downstream analyses, raw mean fluorescence intensity (MFI) values were log2-transformed. The distribution of log2-transformed cytokine MFI profiles showed remarkable consistency across all 9 time points, with comparable medians (**Figure 4A**). Hierarchical clustering analysis effectively revealed relationships between samples, resulting in distinct clustering patterns among similar time points (**Figure 4B**; **Supplementary Figure S1**).

To investigate cytokines linked with disease severity, Z-scores were computed for the 80 measured cytokines between the patient’s improvement to ‘Extremely Severe A’ (Jan 21) and the average of the preceding eight time points when his health was assessed at ‘Extremely Severe D’ (**Supplementary Table S1**). Cytokines of interest were identified based on absolute z-scores deviating by 1 standard deviation or more from the mean (**Table 2**), enabling the identification of subtle changes in cytokine patterns across time points that may be associated with transitions in health severity from extreme severe D to A. Among 80 cytokines, ten exhibited a Z-score of +1 or more, including HGF (4.80), Leptin (3.61), MIF (3.08), ENA78 (1.37), GROA (1.25), IL2 (1.19), CCL17 (1.10), CCL21(1.04), FGF2 (1.0), and TPO (1.0). Additionally, 14 cytokines showed a z-score value of -1 or less including CCL11 (-2.75), MCP1 (-2.27), IL28A(-2.17), CXCL9 (-2.10), IL5 (-2.08), MIP1D (-2.06), SFAS (-1.90), VEGF (-1.79), CCL27 (-1.71), IL9 (-1.51), IL6 (-1.29), MCP4 (-1.20), IL10 (-1.18), IL4 (-1.11) (**Table 2**; **Supplementary Table S1**). Among these 24 cytokines, only nine exhibited statistically significant changes with an absolute z-score > 1 and p-value <= 0.05. These include HGF (z-score: 4.80, p-value: 1.60E-06), Leptin (z-score: 3.61, p-value: 0.0002), MIF (z-score: 3.08, p-value: 0.002), CCL11 (z-score: -2.75, p-value: 0.005), MCP1 (z-score: -2.27, p-value: 0.023), IL28A (z-score: -2.17, p-value: 0.029), CXCL9 (z-score: -2.11, p-value: 0.035), IL5 (z-score: -2.08, p-value: 0.035), and MIP1D (z-score: -2.06, p-value: 0.039) (**Figure 4C**, **Table 2**). This limited 9-plex cytokine panel effectively distinguished ‘Extremely Severe A’ from ‘Extremely Severe D’ (**Figures 4C, D**). Out of these 14 cytokines, only HGF, LEPTIN, and MIF withstood an FDR adjustment of 10% (**Table 2**; **Supplementary Table S1**).

**Table 2:** Top differentially expressed cytokines between healthiest time point (Extremely severe stage A) and average of prior time points (Extremely severe stage D).

Cytokine	Z score	p-value	fdr
HGF	4.80	1.60E-06	0.0001
LEPTIN	3.62	0.0002	0.01
MIF	3.09	0.00	0.05
EOTAXIN/CCL11	-2.75	0.01	0.12
MCP1/CCL2	-2.27	0.02	0.35
IL28A/IFNL2	-2.17	0.03	0.35
MIG/CXCL9	-2.11	0.04	0.35
IL5	-2.08	0.04	0.35
MIP1D/MIP5/CCL15	-2.06	0.04	0.35
SFAS/TNFRSF6	-1.91	0.06	0.45
VEGF	-1.79	0.07	0.53
CTACK/CCL27	-1.72	0.09	0.57
IL9	-1.51	0.13	0.81
ENA78/CXCL5	1.37	0.17	0.97
IL6	-1.29	0.2	0.97
GROA	1.25	0.21	0.97
MCP4/CCL13	-1.20	0.23	0.97
IL2	1.19	0.23	0.97
IL10	-1.18	0.24	0.97
IL4	-1.12	0.26	0.97
TARC/CCL17	1.11	0.27	0.97
6CKINE/CCL21/EXODUS2	1.04	0.3	0.97
FGF2/FGFB	1.01	0.31	0.97
TPO	1.01	0.31	0.97
HGF	4.80	1.60E-06	0.0001

The color-coded heatmap (**Figure 4D**) shows cytokines with significant changes in plasma levels as the patient’s health improved from Extremely Severe D to Extremely Severe A between June 2017 and January 2021. Key cytokines in this transition, such as HGF, LEPTIN, MIF, IL5, CCL11, CCL2, IL28A, CCL15, are highlighted. The red and blue colors indicate whether the cytokine levels increased or decreased. The change for each cytokine was calculated using a z-score based on log-transformed raw MFI values.

Cytokines were filtered based on an absolute Z-Score of ±1 or more, comparing samples from the healthiest time point (01/18/2021) to the average of the 8 previous time points. Significant differences (denoted in red) between the healthiest and preceding time points were determined using p-values, both before and after adjusting for multiple comparisons (controlling for FDR at 10%).

Analyzing cytokines that demonstrate significant differentiation between the patient’s most improved state in January 2021 and the preceding health states revealed distinctive patterns in cytokine deviations. This differentiation is visually represented in the hierarchical clustered heatmap (**Figure 4D**), where clear clusters of time points emerge, highlighting unique cytokine expression profiles. Moreover, among the top differentially expressed cytokines, some exhibit similar temporal profiles (**Figure 4E**). Notably, MCP1 and CCL11 demonstrate analogous trends over time, albeit at varying magnitudes, both showing a decline in the latest time point. Similarly, HGF and MIF display comparable temporal patterns, exhibiting an increase in the most recent time point. In contrast, MCP1 and CCL11 display opposite trends compared to those observed for MIF and, to a lesser degree, with HGF (**Figure 4E**). This intricate interplay underscores the nuanced dynamics of cytokine regulation during the observed period. The analysis of cytokine levels in relation to disease severity indicated a positive correlation for HGF, LEP, and MIF (increase in plasma cytokine level with health improvement and reduced severity) and a negative correlation for CCL11 (decrease in plasma cytokine level with health improvement) during the transition from extremely severe D to extremely severe A. Additionally, negative correlations were observed for MCP1, IL28A, CXCL9, IL5, MIP1D SFAS, VEGF, and CCL27 with health improvement, although statistical significance (p-value < 0.05) was not achieved (**Figure 4F**; **Supplementary Table S2**).

### 3.5 Gene set enrichment and ingenuity pathway analyses

**Figure 5:**
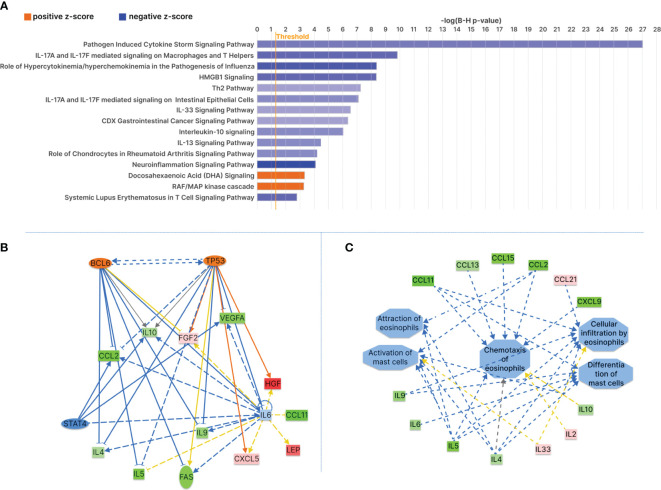
Ingenuity Pathway Analysis of Longitudinal Cytokine Profiling During Health Improvement from Extremely Severe Stage D to A. **(A)** Top significant canonical pathways with an absolute z-score value of 0.8 and B-H p-value ≤ 0.05 are shown. Orange and blue bars represent positive or negative z-scores, indicating predicted pathway activation or inhibition, respectively. **(B)** Upstream regulator analysis indicates the activation of BCL6 and TP53 at the healthiest time point. **(C)** IPA diseases and function analyses predict the inhibition of mast cells and eosinophils functions. Green represents cytokines with reduced plasma levels at the healthiest time points, and red indicates increased cytokines. Orange and blue indicate predicted to be activated or inhibited, respectively.

In exploring the changes in cytokine expression between the healthiest time point and preceding stages, Ingenuity Pathway Analysis (IPA) provided insights into pathways potentially associated with health improvement. Canonical pathway analyses on cytokines with z score value of plus minus 1 or more revealed significant inhibition in several pathways, notably the pathogen-induced cytokine storm signaling pathway (-log (B-H p-value)= 27; z-score = -1.15) (**Supplementary Table S4**), neuroinflammation (-log (B-H p-value)= 4.1; z-score = -2.24), HMGB1 (High Mobility Group Box 1) signaling (-log (B-H p-value)= 8.36; z-score = -1.34), and Systemic Lupus Erythematosus in T cell signaling pathway (-log (B-H p-value)=2.78; z-score: -1.34) (**Figure 5A**; **Supplementary Table S3**). The TH2 pathway also exhibited inhibition (-log (B-H p-value) = 5.5; z-score = -1), aligning with the observed reduction in IL4, IL5, and IL9. Notably, DHA (Docosahexaenoic Acid) signaling (-log (B-H p-value) =3.2; z-score = 1) and RAF/MAP kinase cascade (-log (B-H p-value)=3.26, z-score=1) were predicted to be activated (**Supplementary Table S3**). Canonical pathways analysis on differentially expressed cytokines that passed both the z score value of 1 and more and p value of <0.05 also suggests inhibition of pathogen induced cytokine storm, and IL17 signaling (**Supplementary Figure S2**).

Upstream regulators analyses indicated predicted inhibition in TNF (activation Z score=-1.6, p-value of overlap=2.56E-27) (**Supplementary Figure S3A**), TLR4 (Toll-like receptor 4) (activation Z score=-2.2; p-value of overlap=5.18E-10), PLA2G10 (Phosphatidylcholine 2-Acylhydrolase 10) (**Supplementary Figure S3A, B**) (activation Z score=-2.4, p-value of overlap=5.22E-10), CCL11 (activation Z score=-1.9; p-value of overlap=5.75E-21) (**Supplementary Figure S3B**) (**Supplementary Table S6**), KITLG (KIT proto‐oncogene ligand) (activation Z score=-2.2, p-value of overlap=1.85E-10), IL9 (activation Z score=-2.4, p-value of overlap=2.92E-10), IL18 (activation Z score=-2.2; p-value of overlap=2.65E-17) and NFkB-RelA (activation Z score=-2.0; p-value of overlap=1.96E-10) (**Supplementary Table S5**). Notably, BCL6 (activation Z score=2.1; p-value of overlap=5.97E-11) (**Figure 5B**), EPO (Erythropoietin) (activation Z score=1.99; p-value of overlap=1.29E5), TP53 (activation Z score=1.6; p-value of overlap=1.48E-9) (**Figure 5B**), HBB (hemoglobin subunit beta) (activation Z score=1.9; p-value of overlap=1.68E-18), IL11 (activation Z score=1.96; p-value of overlap=1.02E-6), and SCGB1A1 (Secretoglobin Family 1A Member 1) (activation Z score=1.9; p-value of overlap=1.51E-8) were predicted to be activated (**Supplementary Table S5**, **Figure S3**). Additionally, IPA disease and function analysis predicted reduced activity in mast cell activation (activation z-score=-1.99; overlap p-value=1.44E-10) and mast cell differentiation (activation z-score=-2.23; overlap p-value= 9.01E-11) (**Figure 5C**). There was also a decrease in the attraction of eosinophils (activation z-score=-2; overlap p-value=6.20E-11) and chemotaxis of eosinophils (activation z-score=-1.5; overlap p-value=4.21E-20), as well as cellular infiltration by eosinophils (activation z-score=-1.5; overlap p-value=1.98E12).

### 3.6 Patient blog post sentiment analysis

**Figure 6:**
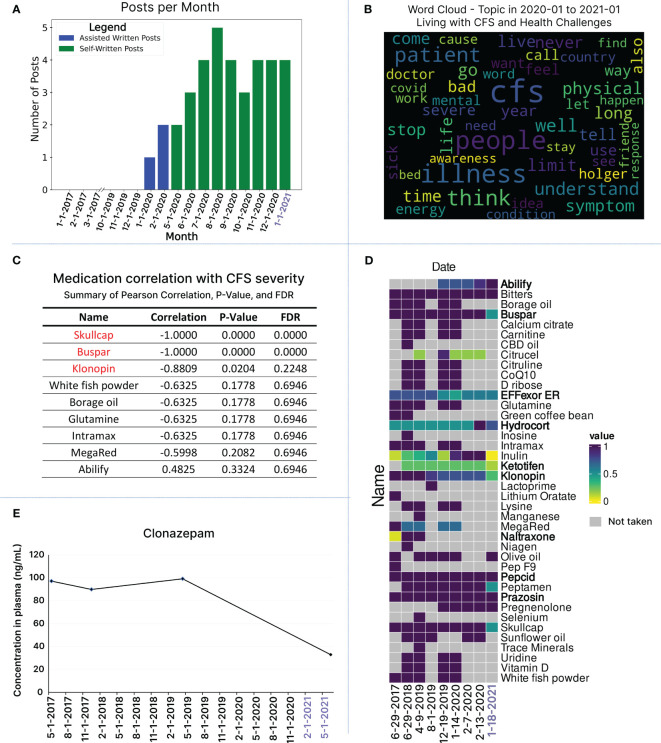
Integrating Longitudinal Cytokine Profiling with Health, Textual, and Medication Data in Relation to Health Improvement. **(A)** Number of blog posts written monthly from 2017 up until the end of January 2021. For the first two months of 2020, the patient had assistance with writing blog posts for the months of January and February (blue bars). From May 2020 and onwards, the patient was feeling well enough to start writing the blog posts on his own (green bars). **(B)** Topic analysis word cloud for blog posts written from January 2020 until the end of January 2021, offering a visual representation of the most frequently occurring words from blog posts in this time frame, all under the topic umbrella of “Living with CFS and health challenges”. **(C)** Correlation analysis of the change in medication and health state over time. Changes in dosage for Skullcap, Buspar and Klonopin showed a strong correlation with improved health. **(D)** Medication usage over time is visualized for the 9 time points, which overlap with cytokine sample time points. To facilitate comparison, the dosage of each medication has been standardized over time using min-max scaling. This standardization is represented in a heatmap, gray squares represent no medication dosage recorded. List of medications are provided in bold to be distinguished from supplements. **(E)** Longitudinal monitoring of Clonazepam concentration in blood, which followed the reduction trend in his intake from 24 mg to 7 mg.

For nuanced insights into subtle health changes, we employed natural language processing to gauge social media activity as a tool for assessing the patient’s functional capacity in daily life activities and obtaining insights into temporal posting trends. Exploratory data analysis focused on post frequency and the average number of words per post at monthly and yearly intervals, revealing no activity from June 2017 to December 2019 (**Figure 6A**). In January and February 2020, the patient received assistance to activate his personal blog and share one post every month (**Figure 6A**). From May 2020 onward, his sensorial intolerance improved to a degree that allowed him to use social media and post blogs without assistance (**Figure 6A**). Throughout the patient’s blog posting journey in 2020 and 2021, there is an increase in the number of posts as well as the length of posts (**Supplementary Figure S4B**). To assess the emotional tone of blog posts (for the years 2020 and 2021), we conducted a sentiment analysis using a pre-trained BERT language model (**Supplementary Figure S4C**). Sentiments were visualized monthly and yearly as negative, neutral, and positive (**Supplementary Figure S4C**). Fisher exact tests revealed significant differences in sentiment distribution (**Supplementary Table S7**). In 2021, the odds ratio for negative vs. positive sentiments was substantially lower (0.13, p=0.03), indicating a significant decrease in negative sentiments. Similarly, neutral vs. positive comparison showed a significant decline in neutral sentiments in 2021 (0.18, p=0.04), emphasizing a notable shift toward a more positive emotional tone over time (**Supplementary Figure S4C**, **Table S7**).

To explore the nuances of linguistic patterns, we conducted a word cloud analysis, visually representing word frequencies across all posts in 2020 and 2021 (**Supplementary Figure S4A**). The most recurring words in 2020 posts include ‘CFS,’ ‘people,’ ‘illness,’ ‘way,’ ‘I’m,’ ‘think,’ ‘see,’ ‘don’t,’ ‘I’ve,’ ‘every,’ ‘life,’ and ‘help.’ In 2021, the frequently used words are ‘CFS,’ ‘dream,’ ‘sleep,’ ‘I’m,’ ‘love,’ ‘long,’ ‘new,’ ‘way,’ ‘day,’ ‘right,’ ‘adventure,’ and ‘people’ (**Supplementary Figure S4A**). While there are overlaps, distinct words and themes emerge when comparing the most used words in 2020 versus 2021. Intrigued by the growing use of LLMs like chatGPT, we informally analyzed the frequently used words (23) identified by the word cloud. We prompted chatGPT to infer the overall message the patient conveyed in his blog posts between Jan 2020 to Jan 2021 (**Figure 5**S).

ChatGPT analysis revealed that the patient reflects on his struggles with ME/CFS, its severity and profound impact on individuals’ lives. Additionally, it suggests the patient’s strong interest in disseminating information and sharing personal experiences related to ME/CFS, thereby contributing to ongoing research efforts (**Supplementary Figure S5**). Promoted for a comparison between 2020 and 2021, chatGPT identifies that although there is still some mix of challenging emotions and experiences, it does note a change in emotional state—a positive outlook in 2021 (**Supplementary Figure S6**).

For a more formal analysis aimed at identifying specific themes across blog posts in 2020 and 2021, we conducted a topic modeling analysis using a latent Dirichlet allocation (LDA) model. Across all posts, the first prominent topic involves words discussing living with CFS and the health challenges the patient faces. Three other main topics identified include personal growth and lifestyle changes, research funding and scientific review, and health strategies and supplements (**Figure 6B**; **Supplementary Figure S4D**). Utilizing natural language processing techniques for textual data analysis saves time and resources compared to manually going through each blog post to identify a patient’s current journey and health state. Furthermore, this reduces bias and interpretation of content, particularly crucial in ME/CFS, where subjective experiences vary. Extracting valuable information from written expressions can provide a means for non-verbal patients to contribute to research and connect with the ME/CFS community. To this end, we developed LexiTime, an application for textual data processing including sentiment analysis and topic modeling. The application is accessible at https://github.com/singjc/lexitime.

Additionally, we explored the relationship between medication changes and the patient’s health status over time (**Figure 6C**; **Supplementary Table S8**). Working closely with caregivers, we gathered a comprehensive medication list spanning the dates of the 9 longitudinal blood samples, including three days before and after each time point. To facilitate comparison, we standardized medication dosages using min-max scaling and presented the patterns in a heatmap, showcasing both individual time points and 7-day windows surrounding each blood draw date (**Figure 6D**; **Supplementary Figure S7**). Our correlation analysis focused on comparing the doses of all medications on the day of blood draw (**Figure 6D**). Significantly, changes in dosage and reduction of intake for Skullcap, Buspar, and Klonopin exhibited a strong correlation with improved health (**Figure 6C**). We also noted a positive correlation between the increase in low-dose Abilify and health improvement, although the latter did not achieve standard significance (**Figure 6C**). The integration of clinical data unveiled a reduction in blood Klonopin levels in response to lowering the medication dose (**Figure 6E**).

## 4 Discussion

**Figure 7:**
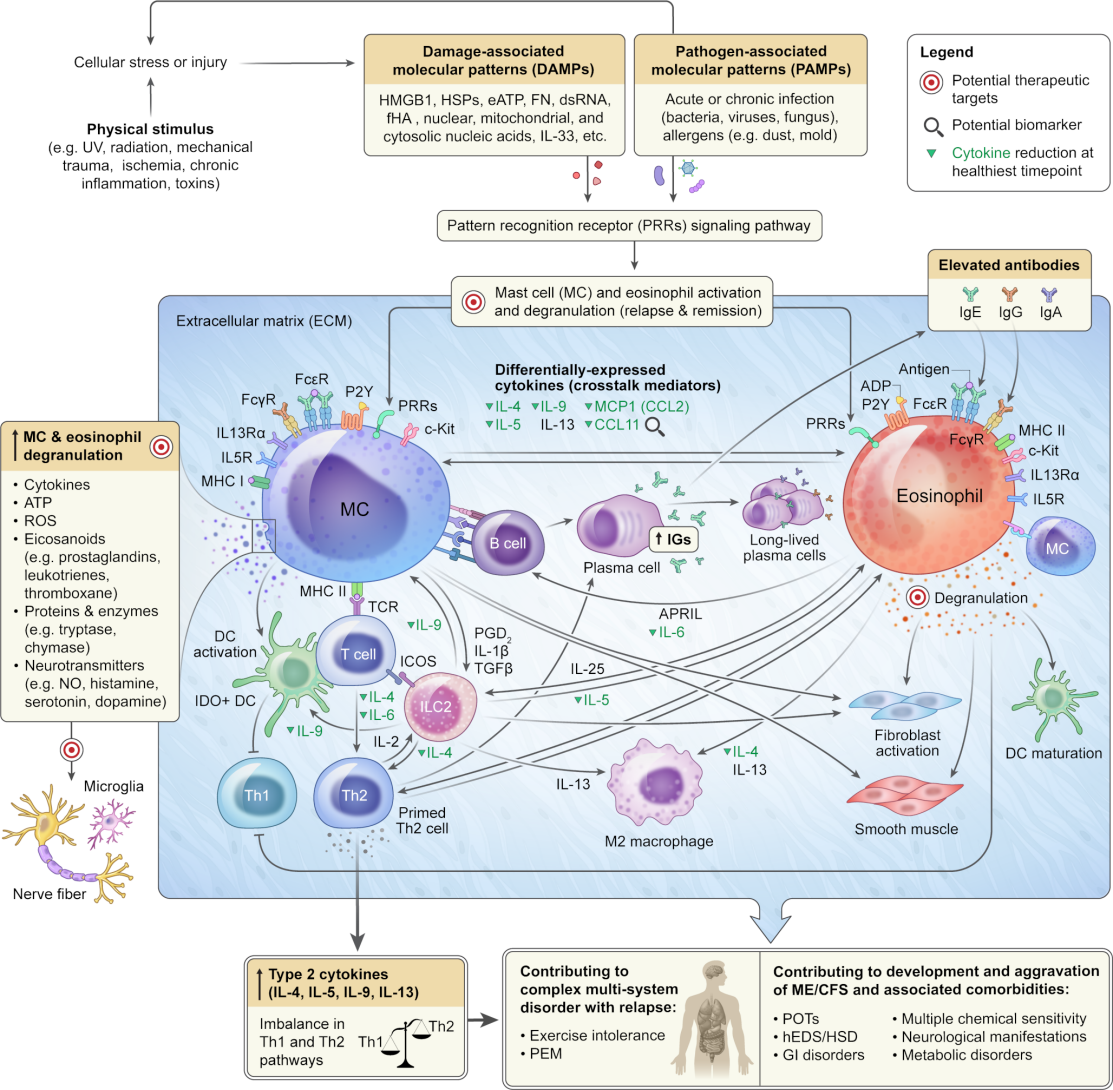
A Potential Mechanism Underlying ME/CFS Development, Aggravation and Comorbidities. Mast cells, present in nearly all human tissues, and eosinophils, found in the gastrointestinal tract, secondary lymphoid tissues, adipose tissue, thymus, mammary gland, and uterus, are tissue-resident cells. Aberrant DAMPs and PAMPs signaling cascades can lead to systemic overactivation and degranulation of mast cells and eosinophils, resulting in the release of over a hundred molecules, including potent inflammatory mediators, into the extracellular matrix of connective tissue. The synergistic activity of mast cells and eosinophils upon systemic activation can skew Th1/Th2 to Th2-immune responses, leading to tissue injuries, autoimmunity, impairment of multiple organs and biological systems as well as causing exercise intolerance and post-exertional malaise in predisposed individuals. Unresolved systemic mast cell and eosinophil overactivation could contribute to the development and aggravation of ME/CFS and related multisystem disorders and comorbidities. The schematic also depicts potential therapeutic targets and biomarkers.

In the ME/CFS research landscape, the focus has traditionally been on comparing patients with healthy controls, with limited attention to longitudinal aspects and periods of improvement (58, 59). Our study addresses this gap by taking a single patient-centered approach (60, 61) exploring molecular and phenotypic differences underlying severity improvements in ME/CFS. While acknowledging the N-of-1 nature of our study and the necessity for larger cohort studies, our integrative longitudinal strategy, incorporating health, clinical, cytokine profiling, and textual data over more than 4 years period offers invaluable insights. These findings enhance our comprehension of factors influencing ME/CFS development and severity, providing personalized insights into medical interventions (**Figure 7**).

We refined the severity framework by introducing the “extremely severe” category, subdivided into A, B, C, D, and E, to capture the complete spectrum of ME/CFS severity and its true debilitating nature at maximum severity (9). While the current very severe category, such as those offered by ICC, includes individuals who are bedridden and require assistance with basic functions (5, 9), some ME/CFS patients may experience extreme sensory sensitivities and post-exertional malaise (PEM) that dangerously affect their ability to receive life necessities such as food and medication. In extreme cases, patients become fully isolated from others, resembling conditions of solitary confinement (18). Recognizing the existence of such a level of severe hypersensitivity and PEM is crucial for advancing care for patients at the highest level of the severity spectrum.

The “extremely severe” category, as outlined in our manuscript, pertains to individuals whose lives are impacted to an extent of 80-100%, profoundly restricting their basic movements, self-care, occupation, communication, and access to social media. It is noteworthy that the integration of the internet and social media into daily life activities over recent decades has been significant; hence, the burden of ME/CFS on those whose severity affects basic access to these platforms may profoundly impact their quality of life and contribute to the worsening of their condition. We believe severity based on a patient’s ability to receive life support and their most basic interaction with the outside world via social media warrants explicit recognition to underscore the significant burden faced by these patients and to delineate appropriate medical interventions and support measures.

While current severity assessment platforms offer a solid foundation for recognizing disease severity and patient classification, integrating this aspect into patients’ diagnosis and severity assessment criteria can provide a more refined delineation of the condition’s severity spectrum (62). This addition facilitates the assessment of even minor improvements in response to treatment. Furthermore, delving into potential shared mechanisms between ME/CFS and Hypermobility Spectrum Disorder addresses significant facets of ME/CFS.

Our ME-CFSTrackerApp supports real-time tracking of symptoms, medications, and life events, benefiting physicians and researchers in more precise single-symptom assessment, treatment optimization, triggers and patterns identification, enhanced patient-doctor communication, longitudinal research data, and efficient data sharing. Crucially, the app enables remote real-time monitoring, facilitating proactive interventions, particularly benefiting severe cases (63). The data can be used for careful subject selection and eligibility criteria for clinical trials, targeted therapy and research studies. Additionally, employing natural language processing and open AI tools like ChatGPT, as well as the development of LexiTime application for textual data processing, including sentiment analysis and topic modeling, holds significant implications for ME/CFS research, particularly for patients with extreme severity who may face challenges in verbal communication (64, 65).

The deliberate inclusion of an extremely severe ME/CFS patient in our analysis offers a unique perspective on immune dynamics at critical disease stages, crucial for understanding the full spectrum of ME/CFS and tailoring interventions (18, 66). The identification of two leukocytosis episodes preceding health issues, particularly linked to infectious mononucleosis, aligns with the widely accepted understanding of ME/CFS etiology. This observation supports the notion that specific infections, such as herpes viruses, can act as catalysts for ME/CFS development (67–70). Additionally, recognizing environmental risk factors such as infection, overexertion, toxin exposure and medication side effects as crucial contributors to ME/CFS progression from mild to extremely severe adds significant value. This acknowledgment not only improves our understanding of the disease’s etiology but also directs focused research efforts to explore underlying mechanisms and develop strategies to mitigate illness severity.

The integration of cytokine analysis from nine longitudinal samples, with health, clinical, and textual data provided deeper insights into the immune signature of ME/CFS during transitions in health severity. At the extremely severe A stage, the patient, while remaining fully bedbound and reliant on caregivers for most aspects of personal life, experienced a reduction in cognitive impairment and sensorial intolerance. This improvement allowed the patient to tolerate sounds, engage with music, access the internet, communicate in writing, and be comfortable with people’s presence in the room. The noteworthy decrease in CCL11 during the healthiest time point aligns with previous ME/CFS cytokine studies, indicating a positive correlation between CCL11 and ME/CFS severity or duration (43, 71). CCL11 has been implicated in various diseases, including fibromyalgia (72), osteoporosis (73), metabolic conditions like non-alcoholic fatty liver disease (NAFLD) (74), accelerated aging, and neurodegenerative disorders such as chronic traumatic encephalopathy (75), multiple sclerosis (76), and the chemotherapy and long COVID-related brain fog (77). The consistent research findings revealing a positive correlation between elevated CCL11 levels, and the severity of sensorial intolerance and cognitive impairment underscore the need for further investigation into the significance of this cytokine in the context of ME/CFS and its potential value as a diagnostic and therapeutic biomarker (**Figure 7**).

Additionally, CCL11 plays a crucial role in recruiting eosinophils, implicating it in allergic responses and a shift toward a Th2 immune response and hypersensitivity. Interestingly, most significantly reduced cytokines at the healthiest time point have also been associated with eosinophils and mast cell activation, Th2 immune response and IgE signaling (**Figure 7**). These cytokines include IL5, MCP1, MIP1D, and CXCL9 (78–80). IL-5 is best known for its major roles in meditating eosinophil growth, activation, and survival and also differentiation of B-1 cells into Ig-secreting cells (78, 79, 81, 82). IL5 is expressed by many cell types including Th2 T-cell subsets, gamma delta T cells, basophils, group 2 innate lymphoid cells (ILC2s) and eosinophils (80, 81). Elevated IL5 has been reported in ME/CFS plasma (83) and many inflammatory conditions like eosinophilic gastroenteritis, eosinophilic dermatitis, and allergic reactions.

The observed reduction in MCP1 levels at the healthiest time point is also in line with a potential positive correlation between aberrant Th2 immune response and ME/CFS severity in this individual. MCP1, expressed by various cell types such as monocyte/macrophage, mast cells (84), dendritic cells, eosinophils (85), osteoclasts, osteoblasts, neurons, astrocytes, and microglia, plays a crucial role in recruiting immune cells like monocytes, eosinophils, and mast cells to inflammation sites resulting from tissue injury or infection (86, 87). This intricate involvement positions MCP1 as a key player in the pathogenesis of inflammatory diseases, including psoriasis, rheumatoid arthritis (88), and various neurological conditions such as brain ischemia (89), Alzheimer’s (90), experimental autoimmune encephalomyelitis (EAE) (91), and traumatic brain injury (92, 93). MCP1 elevation was reported in fibromyalgia and linked to insulin signaling impairment in skeletal muscle cells of these patients (83). This alongside recent reports of increased MCP1 plasma levels in ME/CFS (83), suggests a reduction of MCP1 at healthiest time point might be linked to reduced neuroinflammation (94), improved blood-brain barrier permeability (95), diminished neuronal sensitization, and alleviated endothelial dysfunction (96). Adding another layer to its potential significance, MCP1’s influence on the hypothalamus-pituitary-adrenal (HPA) axis (94), a critical regulator of stress responses and immune function, prompts further exploration into its diagnostic and therapeutic potential for ME/CFS.

Moreover, the reduction in CXCL9 (monokine induced by IFN-gamma) at the healthiest time point also underscores the potential involvement of eosinophils, mast cells, and an imbalance in Th1/Th2 immune responses in ME/CFS etiology in our patient (97–100). CXCL9, acting as a ligand for CXCR3 expressed in synovial mast cells and eosinophils, has been associated with inflammation in synovial tissues (100) and rheumatoid arthritis pathogenesis (99). Interestingly, elevated CXCL9 was reported in the cerebrospinal fluid (CSF) of ME/CFS subjects, particularly those with a disease duration of less than 3 years (101), further emphasizing its potential relevance in ME/CFS etiology, in a subset of patients.

Furthermore, the decrease in IL28A, a type III interferon (102, 103), at the healthiest time point could also contribute to alleviating ME/CFS symptom severity by reducing systemic immune dysregulation, and interferon-associated fatigue, mitigating inflammatory response, and restoring Th1/Th2 balance (104), collectively enhancing cognitive function (105). Despite its physiological importance, aberrant IL28A activity has been linked to the promotion of inflammation and autoimmunity (106), and is associated with conditions such as lupus (107) and post-traumatic sepsis (108).

At the extremely severe stage A, elevation in plasma levels of Leptin, HGF, and MIF correlated positively with reduced disease severity, particularly in sensorial intolerance and cognitive function. It’s essential to acknowledge that the observed cytokine elevation at the healthiest time point may be influenced by factors beyond health improvement, such as extended fasting hours, medication, infection, or trauma. However, considering the diverse multisystem biological functions of those cytokines in maintaining homeostasis, including the central and peripheral nervous system, energy regulation, immune system, and blood circulation, their elevation could contribute to overall health improvement.

Elevated leptin may positively impact ME/CFS by enhancing mental clarity, focus, mood, emotional well-being, metabolic regulation, energy production and utilization, neuroendocrine hormonal balance, motivation and immune function (109–114). Our current findings, indicating a negative correlation between plasma leptin and disease severity, differ from previous studies (42, 43), potentially attributed to the longitudinal nature of our research and variations in severity scales. Furthermore, the observed rise in leptin levels in the latest time point may be associated with slight changes in BMI (41, 115).

The varied physiological role of HGF (hepatocyte growth factor) suggests that an increase level may reduce ME/CFS symptom severity through tissue repair (116), anti-inflammatory effects, enhanced energy metabolism (117), neuroprotection (118, 119), improved blood circulation (120), immune modulation (121), and antioxidant properties (122–124). Further research into HGF’s role in ME/CFS is warranted due to inconsistencies across studies (117–121).

MIF is a multifunctional molecule produced by various cell types, including activated macrophages. Beyond its proinflammatory roles, MIF is associated with many pathways such as inflammation, neural activities, and neuroplasticity (125, 126). It regulates catecholamine metabolism (127), protects dopaminergic neurons and has antidepressant effects at high concentrations. Additionally, MIF impacts the hypothalamic−pituitary−adrenal cortex axis (125, 126). Elevated MIF may correlate with improved ME/CFS symptoms, particularly in cognitive function and sensorial intolerance. However, elevated MIF at the healthiest point may stem from factors like infection (128), glucocorticoid use (129), and trauma (130). Further investigation is needed to understand MIF’s role in ME/CFS pathophysiology, disease severity, and prognosis.

While specific cytokine changes, including the reduction in SFAS/FASLG, VEGF, CTACK/CCL27, IL9, IL6, MCP4, IL10, IL4, and increases in ENA78, GROA, IL2, TRACE, CCL21, FGF2, IL33, and TPO, did not achieve statistical significance individually, their collective alterations, as indicated by Z scores, may reveal a significant impact on the cytokine landscape in ME/CFS. Notably, IL4, IL6, IL9, IL10 (131), and IL33 play crucial roles in Th-2 immunity, the mast cell and eosinophil signaling network (132–138) (**Figure 7**).

Canonical pathway and upstream regulator analysis suggests potential mechanisms which could attenuate mast cells and eosinophil activities at the healthiest time point. This involves the predicted activation of Docosahexaenoic acid (DHA) and inhibition of KITLG and TLR4 signaling pathways (139). DHA and its metabolite, docosahexaenoyl ethanolamide (DHEA) show promise in preventing mast cell degranulation, IgE-mediated anaphylaxis reaction (140), eosinophil dysfunction (141) and also facilitates synaptogenesis and synaptic activity (similar to the endogenous cannabinoid receptor ligand anandamide) (142). Inhibition of KITLG and TLR4 could also decrease levels of various cytokines, including IL10, IL4, IL5, IL6, CCL2, and MCP1, thereby mitigating mast cell and eosinophil activation and aberrant Th2 immune responses (140, 143–149). Targeting TLR4 holds promise for reducing neuroinflammation (150–152).

Our study unveils novel insights into the potential roles of TP53 (tumor protein p53) and BCL6 (B-cell CLL/lymphoma 6) in ME/CFS pathogenesis (153, 154). Notably, both BCL6 and TP53 function as negative regulators in IgE-mediated mast cell activation (155, 156), exerting a dampening effect on both early and late-phase anaphylaxis. BCL6, a master regulator of humoral immunity, negatively modulates key molecules and cells associated with Th2-type inflammation (156–158). The implications of its involvement in preventing or attenuating allergic diseases suggest a potential link to ME/CFS in a subset of patients, especially considering its role in experimental autoimmune encephalomyelitis (EAE) (156). TP53, beyond its role as a tumor suppressor, plays a crucial part in various physiological processes, including cell metabolism, mitochondrial respiration, autophagy, and stress response. Balanced TP53 activation could reduce ME/CFS severity by promoting cellular repair (159), regulating metabolic pathways (160), and mitigating inflammation through the suppression of NF-κB transcriptional activity and mast cells and eosinophil-mediated Th2 dominant response (161). TP53’s role in neurite outgrowth suggests potential benefits for cognitive function and overall neurological health (162, 163). These findings underscore the imperative for further exploration into the involvement of TP53 and BCL6 in ME/CFS pathophysiology.

The identification of a diminished Th2 immune response, coupled with reduced mast cell and eosinophil activation at the healthiest time point, unravels potential underlying pathological mechanisms in ME/CFS. This observation holds particular relevance to its association with major comorbidities such as EDS/hEDS/HSD. Mast cells and eosinophils, widely distributed in connective tissues (164), play crucial roles in immune regulation and extracellular matrix homeostasis (165, 166).

Notably, mast cell activation syndrome (MCAS) is highly prevalent in ME/CFS and connective tissue disorders, including EDS/hEDS/HSD. Additionally, aberrant eosinophil function is commonly observed in connective tissue disorders (167, 168) and reported in the context of ME/CFS (169, 170). This underscores the necessity for further exploration into the dynamic interactions between mast cell and eosinophil immune responses and connective tissue function in ME/CFS (**Figure 7**).

Both mast cells and eosinophils exhibit widespread tissue distribution, with mast cells present in nearly all human tissues (171), and eosinophils mainly localized in the gastrointestinal tract, secondary lymphoid tissues, adipose tissue, thymus, mammary gland, and uterus (172). These cells play indispensable roles in both innate and adaptive immunity, particularly in tissues closely exposed to environmental factors like the skin and intestinal lining (173, 174), where they are engaged in bidirectional mast cell–eosinophil interactions known as the allergic effector unit (AEU) (175, 176). Infections, allergens (177), and exposure to environmental risk factors (e.g., UV, radiation, stress, mechanical trauma, and hypoxia) can activate a wide array of receptors, including PRRs (pattern recognition receptors) (e.g., TLRs, NLRs, RLRs), FC receptors, P2Y receptors (P2YR) (178, 179), MHC (major histocompatibility complex) class II (180, 181) and complement receptors (C3a and C5a) (182–184) on mast cells and eosinophils via binding to their cognate ligands. These factors could also lead to cell/tissue injury, resulting in the release of endogenous danger signals or DAMPs (damage-associated molecular patterns) (e.g., HMGB1, HSPs, eATP, FN, dsRNA, fHA, nuclear, mitochondrial, and cytosolic nucleic acids, cytokines) (185) into the extracellular space. Here, they can engage with the same PRRs to initiate mast cell (135) and eosinophil (186, 187) activation and intricate crosstalk between these cells and the rest of the immune and non-immune cells (137, 138, 180, 181, 188). Upon activation, both cells undergo degranulation, releasing various inflammatory mediators, including reactive oxygen species (ROS), cytokines, eicosanoids (e.g., prostaglandins, leukotrienes, thromboxane), proteins (cationic proteins) and enzymes (e.g., tryptase, chymase, peroxidase, β-hexosaminidase, β-glucuronidase, arylsulfatases) as well as neurotransmitters (e.g., nitric oxide, histamine, serotonin, dopamine, substance P) (188–190) (**Figure 7**).

Due to their widespread distribution, the overactivation of mast cells and eosinophils can impact multiple biological systems and organs, affecting cardiovascular, endothelial (191, 192), epithelial, mucosal, microvascular (193), metabolic (194), muscular, gastrointestinal, and connective tissues (193, 195–197) as well as peripheral, central and autonomic nervous system. This cascade of effects can contribute to the development of complex multi-system conditions, exemplified by ME/CFS, which manifests with a diverse range of comorbidities (198) such as connective tissue disorders, small fiber neuropathy, migraine (199–201), POTS (202, 203), immune system hypersensitivity (204, 205), and dermatological manifestations (e.g., dermatitis, tingling or numbness, sensitivity, and allodynia) (206), as well as neuroinflammation, GI disorders, leaky gut (207), and autoimmunity observed in many patients (82, 143, 208–213). The excessive response of mast cells and eosinophils, coupled with aberrant degranulation, not only has the potential to trigger or exacerbate congenital conditions but also contributes to the development of acquired forms (214). This aspect is particularly relevant in conditions like hEDS and HSD, where underlying genetic variations remain unidentified (215, 216).

While mast cells and eosinophils have been traditionally associated with type 2 immunity during allergies and helminth infections, they actively participate in immune responses against various pathogens, including bacteria, fungi, and viruses (174, 217–220). Their capacity to survey the environment through numerous receptors enables them to finely tune a balance between immune activation and suppression. This orchestration involves intricate crosstalk with a variety of cells, including dendritic (221), B, T, type 2 innate lymphoid cells (ILC2) (222), macrophage, monocyte, fibroblast, smooth muscle, neurons, microglia (223), as well as epithelial and endothelial cells. Recent revelations highlight the dual nature of mast cells and eosinophils (175, 182, 224, 224). While acting as potent agents against helminths, they also play a surprising role in fostering the coevolution of helminth parasites with their hosts through immunosuppressive activities (225). A noteworthy mechanism involves the reduction of Th1 immune cell proliferation and the promotion of apoptosis via the indoleamine 2,3-dioxygenase (IDO) gene (226). This leads to a decrease in Th1 cells and a tilting of the immune balance from Th1/Th2 towards Th2-dominated responses (227–233), potentiating systemic autoimmune inflammatory diseases (229).

Research indicates that overtraining can shift the Th1 to Th2 phenotype, resulting in diminished Th1 and NK cell function (234). Lower NK cell levels have been reported among ME/CFS patients (235). Furthermore, an overactive Th2 immune response has been implicated in individuals with exercise intolerance (234, 236). These findings, suggest an intricate crosstalk between dysregulated immune system, exercise intolerance, and post-exertional malaise in ME/CFS. Additionally, these findings might imply a primed Th2 immune system plays a role in the adverse reactions observed in ME/CFS patients to high-intensity aerobic exercise. Recent findings linking elevated Th2 immune response in long COVID patients with adverse reactions to aerobic exercise further highlight the importance of investigating the Th1/Th2 axis imbalance and post-exertional malaise in ME/CFS and related comorbidities for insights into etiology and lifestyle adjustments (236).

Adding to their significance, mast cells and eosinophils play pivotal roles in adaptive immunity, and guiding T (157, 237, 238) and B cells to transform into effector cells, such as antibody-producing plasmablasts and long-lived plasma cells (158, 189, 239–246). Their activated states may contribute to aberrant elevated antibody production (247) and T and B cell-dependent inflammatory and autoimmune diseases. The surplus IgEs, IgAs, and IgGs binding to mast cell and eosinophil FC receptors set off a vicious cycle, amplifying degranulation and escalating tissue damage and promoting autoimmunity (248–250). Uncontrolled Th2 activity may also mitigate pathogenic T-cell immunity. With elevated antibody levels observed in many ME/CFS patients (251–253), delving into the role of mast cell and eosinophil activation in triggering abnormal antibody responses could provide deeper insights into ME/CFS pathogenesis, severity, and guide diagnostic and therapeutic strategies (254).

In our study analyzing medication data from June 2017 to January 2021, a positive correlation was found between health improvement in ME/CFS patients and a slight increase in low-dose Abilify (Aripiprazole), alongside the reduction and optimization of doses for Skullcap, buspar (buspirone), and klonopin (clonazepam). All immune cells, including mast cells and eosinophils, express receptors for various neurotransmitters and neuropeptides (255–258). Optimizing the dose of these medications, known for modulating neurotransmitters and neuropeptides, may contribute to health improvement by modulating the central, and peripheral nervous system and restoring Th1/Th2 balance, mitigating mast cells (259, 260) and eosinophils’ overactivation (261, 262) along with other relevant biological players (255–258) (**Figure 7**).

Abilify’s acts on dopamine D2 and serotonin 5-HT1A receptors, as a partial agonist and antagonist, respectively (263). As a partial agonist, Abilify could act as a D2 agonist, increasing dopamine activity in the presence of low levels of endogenous dopamine and exerting antagonistic activity in the presence of high levels (263). An in-depth investigation into aberrant dopaminergic signaling in ME/CFS and Abilify’s use is warranted, given varying anecdotal reports, some indicating benefits and others worsening symptoms (264). Klonopin binds to GABA receptors in and outside the brain (265–269), enhancing GABA’s inhibitory effect and regulating serotonin utilization (270, 271). Buspirone, an anxiolytic drug, acts as a partial agonist of serotonin and dopamine receptors (272). Despite distinct targets, both klonopin and buspar have been used to manage pain and improve cognition (273–277). However, higher doses may result in cognitive issues (277). Skullcap, a native American medicinal plant has been used for treating menstrual disorders, nervousness, and kidney problems (278). However, at higher doses it can cause giddiness, stupor, mental confusion, twitching, irregular heartbeat, and seizures (279). Notably, baicalin, a component of Skullcap, binds to GABA A receptor. Optimizing the doses of these medications could potentially enhance cognitive function and minimize side effects due to drug interactions (280–283). Our analysis of medication correlations with severity underscores challenges faced by ME/CFS patients in navigating their path to recovery. The intricate and diverse nature of ME/CFS, coupled with the absence of FDA-approved treatments, often prompts patients to adopt a trial-and-error approach, experimenting with various medications and interventions to address their health needs (284, 285).

Potential interactions among these interventions are frequently overlooked due to the absence of proper diagnostic assessments and objective evaluations of disease severity. Developing tools to monitor and assess potential adverse effects will enhance patient safety, inform personalized treatment plans, and contribute to the advancement of ME/CFS care (170). The ME/CFSTracker app serves as a valuable tool for patients, caregivers, and medical teams, enabling the easy collection of longitudinal health changes and a more precise assessment of severity in response to therapeutics and nutraceutical interventions.

Our study has limitations, such as a small sample size and the heterogeneous nature of ME/CFS, which may limit the generalizability of our findings. The observed aberrant Th2-cytokine expression and dysregulated mast cells and eosinophil function, causing a shift from Th1 to Th2 immune response, may apply to a specific subset of patients rather than the entire spectrum (286–290). Further longitudinal studies are needed to validate and extend the scope of our results.

## 5 Conclusion

Our study underscores the values of integrating longitudinal health, clinical, pharmaceutical, nutraceutical, and multi-omics data, and the use AI to advance our understanding of factors and mechanisms underlying ME/CFS development, progression, and its associated comorbidities, guiding personalized data-driven therapies (279). We introduced an updated platform for objective severity assessment and created two applications, the ME-CFSTrackerApp, and LexiTime, aiming to facilitate real-time symptom tracking and text mining to optimize treatment strategies and communication. Our longitudinal cytokine profiling suggests the need for further research into the role of aberrant Th2-type cytokines and the synergistic activities between mast cells and eosinophils in the pathogenesis and severity of ME/CFS, particularly in cognitive impairment and sensorial intolerance. Our results also highlight a potential shared underlying mechanism between ME/CFS and its major comorbidities such as hEDS/HSD, POTS, MCAS, multiple chemical sensitivity, peripheral neuropathy, and the neuro-immune and brain-gut interaction axis.

Our findings align with previous research, supporting the potential of CCL11, IL5, and MCP1 as biomarkers for ME/CFS diagnostics and therapeutics. Identification of master regulators like BCL6, TP53, KTLG offers a mechanistic model linking chronic systemic activation of mast cells and eosinophils to the development and aggravation of multi-system conditions such as ME/CFS (30, 160, 196, 202, 280–282). Our results underscore the need for further exploration into mast cell- and eosinophil-directed biologic therapies, and the significance of low-dose drugs with partial agonists activity toward neurotransmitters in ME/CFS treatment (**Figure 7**).

## Data availability statement

The original contributions presented in the study are included in the article/**Supplementary Material**, further inquiries can be directed to the corresponding author/s.

## Ethics statement

The studies involving humans were approved by Stanford Research Compliance Office. The studies were conducted in accordance with the local legislation and institutional requirements. The participants provided their written informed consent to participate in this study. Written informed consent was obtained from the individual(s) for the publication of any potentially identifiable images or data included in this article.

## Author contributions

FJ: Conceptualization, Data curation, Formal analysis, Funding acquisition, Investigation, Methodology, Project administration, Resources, Supervision, Validation, Visualization, Writing – original draft, Writing – review & editing. JS: Formal analysis, Methodology, Software, Visualization, Writing – original draft, Writing – review & editing. RM: Data curation, Methodology, Visualization, Writing – review & editing. SJ: Data curation, Writing – review & editing. JD: Data curation, Writing – review & editing. WD: Data curation, Writing – review & editing. NJ: Methodology, Software, Writing – review & editing. KW: Methodology, Software, Writing – review & editing. AR: Visualization, Writing – review & editing. HR: Writing – review & editing. HM: Writing – review & editing. MS: Funding acquisition, Resources, Writing – review & editing. RD: Funding acquisition, Resources, Writing – review & editing.
