# Supplementary material for: Longitudinal cytokine and multi-modal health data of an extremely severe ME/CFS patient with HSD reveals insights into immunopathology, and disease severity
Source: Front Immunol. 2024 Apr 8;15:1369295. doi: 10.3389/fimmu.2024.1369295 (PMC11033372; doi:10.3389/fimmu.2024.1369295)
Supplement: Supplementary file 1 [file DataSheet_1.docx]

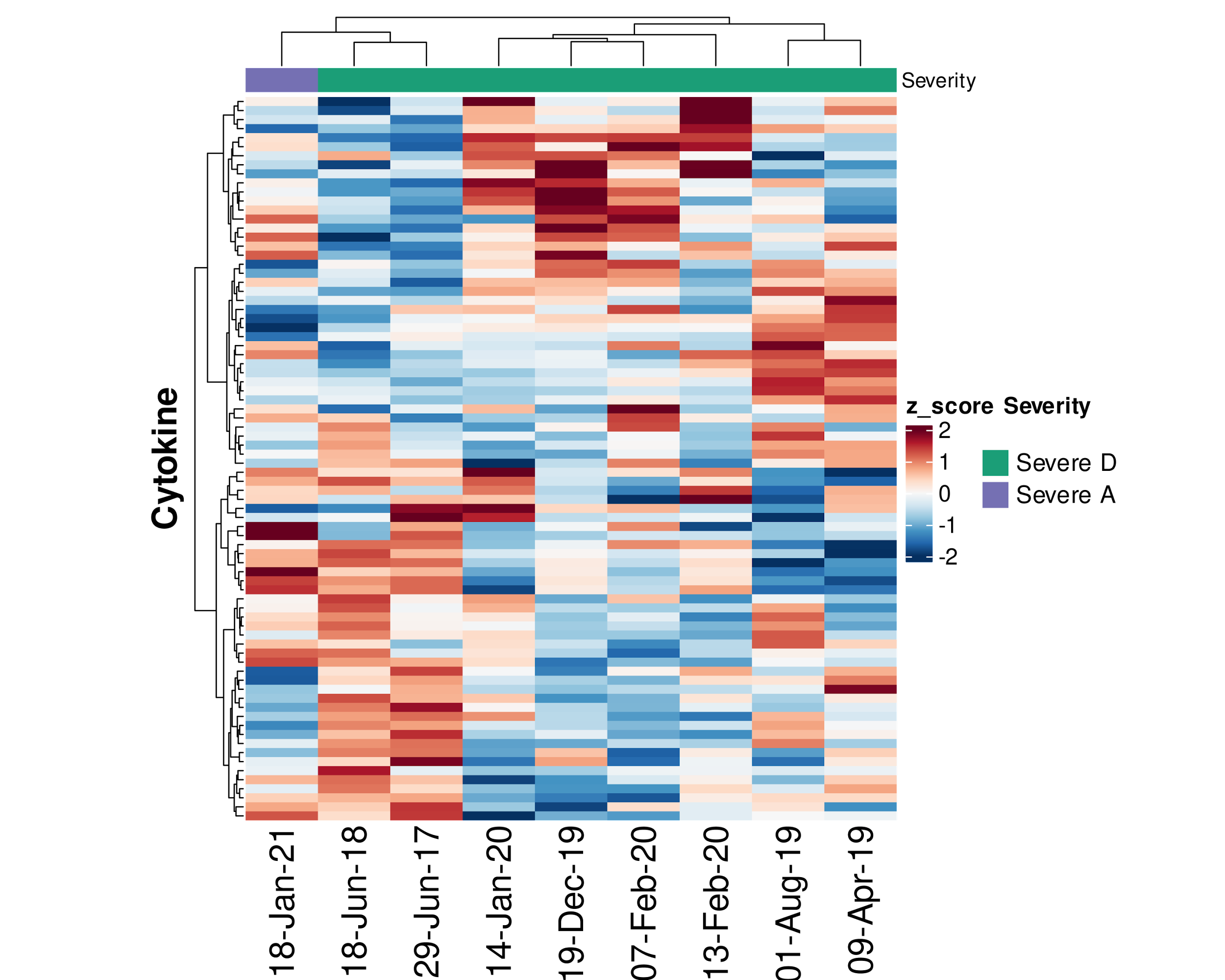


**Figure S1.** Heat map illustrating the normalized (z-score) mean fluorescence intensity (MFI) of the 80 measured cytokines in 9 longitudinal blood samples from an extremely severe ME/CFS patient. Both rows and columns are sorted based on hierarchical clustering. The x-axis is labeled by time-point, color-coded based on z-scores computed between the last time point (Jan 21) when the patient's severity improved to 'extremely severe A' and the preceding eight time points. Dark blue indicates the lowest z-scores, while dark red indicates the highest.

**Table S1.** Z-Scores Values Computed Between the Patient's Healthiest Time Point (Jan 21) and the Preceding Eight Time Points.

| Variable | Z-Score | P-Value | FDR |
| --- | --- | --- | --- |
| HGF | 4.80 | 1.60E-06 | 0.0001 |
| LEPTIN | 3.62 | 0.00 | 0.01 |
| MIF | 3.09 | 0.00 | 0.05 |
| EOTAXIN/CCL11 | -2.75 | 0.01 | 0.12 |
| MCP1/CCL2 | -2.27 | 0.02 | 0.35 |
| IL28A/IFNL2 | -2.17 | 0.03 | 0.35 |
| MIG/CXCL9 | -2.11 | 0.04 | 0.35 |
| IL5 | -2.08 | 0.04 | 0.35 |
| MIP1D/MIP5/CCL15 | -2.06 | 0.04 | 0.35 |
| SFAS/TNFRSF6 | -1.91 | 0.06 | 0.45 |
| VEGF | -1.79 | 0.07 | 0.53 |
| CTACK/CCL27 | -1.72 | 0.09 | 0.57 |
| IL9 | -1.51 | 0.13 | 0.81 |
| ENA78/CXCL5 | 1.37 | 0.17 | 0.97 |
| IL6 | -1.29 | 0.20 | 0.97 |
| GROA | 1.25 | 0.21 | 0.97 |
| MCP4/CCL13 | -1.20 | 0.23 | 0.97 |
| IL2 | 1.19 | 0.23 | 0.97 |
| IL10 | -1.18 | 0.24 | 0.97 |
| IL4 | -1.12 | 0.26 | 0.97 |
| TARC/CCL17 | 1.11 | 0.27 | 0.97 |
| 6CKINE/CCL21/EXODUS2 | 1.04 | 0.30 | 0.97 |
| FGF2/FGFB | 1.01 | 0.31 | 0.97 |
| TPO | 1.01 | 0.31 | 0.97 |
| IL33/NFHEV(MATURE) | 1.00 | 0.32 | 0.97 |
| IL1RA | -0.98 | 0.33 | 0.97 |
| IP10/CXCL10 | -0.91 | 0.36 | 0.97 |
| IL27 | -0.90 | 0.37 | 0.97 |
| MCSF | -0.86 | 0.39 | 0.97 |
| IL1B | 0.83 | 0.40 | 0.97 |
| MIP1A/CCL3 | -0.80 | 0.42 | 0.97 |
| IL18 | 0.79 | 0.43 | 0.97 |
| IL3 | -0.76 | 0.45 | 0.97 |
| MCP2/CCL8 | -0.74 | 0.46 | 0.97 |
| IL17E/IL-25 | -0.67 | 0.50 | 0.97 |
| IL1A | -0.65 | 0.51 | 0.97 |
| IL8/CXCL8 | -0.64 | 0.52 | 0.97 |
| PAI1/SERPINE | -0.60 | 0.55 | 0.97 |
| SDF1A+B/CXCL12 | -0.59 | 0.55 | 0.97 |
| MDC/CCL22 | 0.58 | 0.56 | 0.97 |
| IL17F | 0.55 | 0.58 | 0.97 |
| IL12P70 | 0.55 | 0.58 | 0.97 |
| GCSF | 0.53 | 0.59 | 0.97 |
| IL17A/CTLA8 | 0.53 | 0.60 | 0.97 |
| IL16 | -0.53 | 0.60 | 0.97 |
| RESISTIN | -0.52 | 0.61 | 0.97 |
| IL7 | 0.48 | 0.63 | 0.97 |
| IL20 | -0.44 | 0.66 | 0.97 |
| EOTAXIN2/CCL24/MPIF2 | 0.42 | 0.67 | 0.97 |
| TRAIL/TNFSF10 | 0.42 | 0.67 | 0.97 |
| GMCSF | 0.41 | 0.68 | 0.97 |
| IL22 | -0.39 | 0.70 | 0.97 |
| FRACTALKINE/CX3CL1 | -0.36 | 0.72 | 0.97 |
| SCD40L | -0.33 | 0.74 | 0.97 |
| FLT3L | -0.33 | 0.74 | 0.97 |
| IFNG | 0.32 | 0.75 | 0.97 |
| TSLP | 0.32 | 0.75 | 0.97 |
| FASL | -0.31 | 0.75 | 0.97 |
| MIP1B/CCL4 | -0.31 | 0.76 | 0.97 |
| PDGFAB/BB | -0.30 | 0.76 | 0.97 |
| BCA1/CXCL13 | 0.30 | 0.76 | 0.97 |
| SICAM1 | 0.30 | 0.77 | 0.97 |
| TNFB/LYMPHOTOXINA(LTA) | -0.29 | 0.77 | 0.97 |
| IFNA2 | -0.27 | 0.79 | 0.97 |
| SVCAM1 | 0.25 | 0.80 | 0.97 |
| IL12P40 | 0.24 | 0.81 | 0.97 |
| IL21 | -0.21 | 0.83 | 0.97 |
| IL13 | 0.21 | 0.83 | 0.97 |
| PDGFAA | -0.19 | 0.85 | 0.97 |
| IL23 | 0.18 | 0.86 | 0.97 |
| TGFA | 0.16 | 0.87 | 0.97 |
| MCP3/CCL7 | -0.16 | 0.87 | 0.97 |
| SCF | 0.14 | 0.89 | 0.98 |
| IL15 | -0.09 | 0.93 | 0.98 |
| EGF | -0.07 | 0.94 | 0.98 |
| TNFA | -0.06 | 0.95 | 0.98 |
| EOTAXIN3/CCL26 | -0.06 | 0.95 | 0.98 |
| I309/CCL1 | 0.03 | 0.97 | 0.98 |
| LIF | -0.03 | 0.97 | 0.98 |
| RANTES/CCL5 | -0.03 | 0.98 | 0.98 |

P-values were generated from two-tailed normal distribution samples from each z-score, followed by FDR p-value adjustment for multiple-hypothesis testing.

**Table S2.** Results of Pearson correlation analysis identifying independent cytokines associated with health improvement.

| Variable | Correlation | P-Value |
| --- | --- | --- |
| HGF | 0.86 | 0.003 |
| LEPTIN | 0.79 | 0.01 |
| MIF | 0.74 | 0.02 |
| EOTAXIN/CCL11 | -0.7 | 0.04 |
| MCP1/CCL2 | -0.63 | 0.07 |
| IL28A/IFNL2 | -0.61 | 0.08 |
| MIG/CXCL9 | -0.6 | 0.09 |
| IL5 | -0.6 | 0.09 |
| MIP1D/MIP5/CCL15 | -0.59 | 0.09 |
| SFAS/TNFRSF6 | -0.56 | 0.11 |
| VEGF | -0.54 | 0.13 |
| CTACK/CCL27 | -0.52 | 0.15 |
| IL9 | -0.47 | 0.2 |
| ENA78/CXCL5 | 0.44 | 0.24 |
| IL6 | -0.42 | 0.26 |
| GROA | 0.41 | 0.28 |
| MCP4/CCL13 | -0.39 | 0.29 |
| IL2 | 0.39 | 0.3 |
| IL10 | -0.39 | 0.3 |
| IL4 | -0.37 | 0.33 |
| TARC/CCL17 | 0.37 | 0.33 |
| 6CKINE/CCL21/EXODUS2 | 0.35 | 0.36 |
| FGF2/FGFB | 0.34 | 0.37 |
| TPO | 0.34 | 0.37 |
| IL33/NFHEV(MATURE) | 0.33 | 0.38 |
| IL1RA | -0.33 | 0.39 |
| IP10/CXCL10 | -0.31 | 0.42 |
| IL27 | -0.31 | 0.42 |
| MCSF | -0.29 | 0.44 |
| IL1B | 0.28 | 0.46 |
| MIP1A/CCL3 | -0.27 | 0.48 |
| IL18 | 0.27 | 0.48 |
| IL3 | -0.26 | 0.5 |
| MCP2/CCL8 | -0.25 | 0.51 |
| IL17E/IL-25 | -0.23 | 0.55 |
| IL1A | -0.23 | 0.56 |
| IL8/CXCL8 | -0.22 | 0.57 |
| PAI1/SERPINE | -0.21 | 0.59 |
| SDF1A+B/CXCL12 | -0.21 | 0.59 |
| MDC/CCL22 | 0.2 | 0.6 |
| IL17F | 0.19 | 0.62 |
| IL12P70 | 0.19 | 0.62 |
| GCSF | 0.19 | 0.63 |
| IL17A/CTLA8 | 0.18 | 0.63 |
| IL16 | -0.18 | 0.64 |
| RESISTIN | -0.18 | 0.64 |
| IL7 | 0.17 | 0.66 |
| IL20 | -0.15 | 0.69 |
| EOTAXIN2/CCL24/MPIF2 | 0.15 | 0.7 |
| TRAIL/TNFSF10 | 0.15 | 0.7 |
| GMCSF | 0.14 | 0.71 |
| IL22 | -0.14 | 0.72 |
| FRACTALKINE/CX3CL1 | -0.13 | 0.74 |
| SCD40L | -0.12 | 0.76 |
| FLT3L | -0.12 | 0.77 |
| IFNG | 0.11 | 0.77 |
| TSLP | 0.11 | 0.77 |
| FASL | -0.11 | 0.78 |
| MIP1B/CCL4 | -0.11 | 0.78 |
| PDGFAB/BB | -0.11 | 0.79 |
| BCA1/CXCL13 | 0.11 | 0.79 |
| SICAM1 | 0.11 | 0.79 |
| TNFB/LYMPHOTOXINA(LTA) | -0.1 | 0.8 |
| IFNA2 | -0.1 | 0.81 |
| SVCAM1 | 0.09 | 0.82 |
| IL12P40 | 0.09 | 0.83 |
| IL21 | -0.08 | 0.85 |
| IL13 | 0.08 | 0.85 |
| PDGFAA | -0.07 | 0.86 |
| IL23 | 0.06 | 0.87 |
| TGFA | 0.06 | 0.88 |
| MCP3/CCL7 | -0.06 | 0.88 |
| SCF | 0.05 | 0.9 |
| IL15 | -0.03 | 0.94 |
| EGF | -0.03 | 0.95 |
| TNFA | -0.02 | 0.95 |
| EOTAXIN3/CCL26 | -0.02 | 0.96 |
| I309/CCL1 | 0.01 | 0.98 |
| LIF | -0.01 | 0.98 |
| RANTES/CCL5 | -0.01 | 0.98 |

Pearson correlation analysis examined the association between cytokine levels and ME/CFS severity from extreme levels D to A. Positive correlation coefficients indicate an increase in cytokine levels with health improvement, while negative values indicate a decrease.

**Table S3.** Ingenuity Pathway Analysis (IPA) Top Canonical Pathways with an Absolute Z-Score Value of 0.8 and B-H p-Value ≤ 0.05

| Ingenuity Canonical Pathways | -log(B-H p-value) | Ratio | z-score | Molecules |
| --- | --- | --- | --- | --- |
| Th1 and Th2 Activation Pathway | 8.29 | 0.04 | #NUM! | IL10,IL2,IL33,IL4,IL5,IL6,IL9 |
| Glucocorticoid Receptor Signaling | 7.35 | 0.02 | #NUM! | CCL11,CCL13,CCL2,IL10,IL2,IL4,IL5,IL6,IL9 |
| Docosahexaenoic Acid (DHA) Signaling | 3.32 | 0.02 | 1.00 | FAS,FGF2,HGF,VEGFA |
| RAF/MAP kinase cascade | 3.26 | 0.02 | 1.00 | FGF2,HGF,IL2,IL5 |
| Th2 Pathway | 7.25 | 0.04 | -0.82 | IL10,IL2,IL33,IL4,IL5,IL9 |
| IL-33 Signaling Pathway | 6.54 | 0.03 | -0.82 | CCL17,CCL2,IL33,IL4,IL5,IL6 |
| CDX Gastrointestinal Cancer Signaling Pathway | 6.36 | 0.03 | -0.82 | IL2,IL33,IL4,IL5,IL6,LEP |
| Differential Regulation of Cytokine Production in Intestinal Epithelial Cells by IL-17A and IL-17F | 7.1 | 0.17 | -1.00 | CCL2,CXCL1,IL10,IL9 |
| Interleukin-10 signaling | 6.02 | 0.09 | -1.00 | CCL2,CXCL1,IL10,IL6 |
| IL-13 Signaling Pathway | 4.45 | 0.03 | -1.00 | CCL11,IL10,IL33,IL4 |
| Role of Chondrocytes in Rheumatoid Arthritis Signaling Pathway | 4.19 | 0.03 | -1.00 | CCL2,IL33,IL6,VEGFA |
| Pathogen Induced Cytokine Storm Signaling Pathway | 27 | 0.05 | -1.15 | CCL11,CCL13,CCL15,CCL17,CCL2,CCL21,CCL27,CXCL1,CXCL5,CXCL9,IL10,IL2,IL33,IL4,IL5,IL6,IL9,LEP,VEGFA |
| Differential Regulation of Cytokine Production in Macrophages and T Helper Cells by IL-17A and IL-17F | 9.83 | 0.28 | -1.34 | CCL2,CXCL1,IL10,IL6,IL9 |
| HMGB1 Signaling | 8.36 | 0.04 | -1.34 | CCL2,IL2,IL33,IL4,IL5,IL6,LEP |
| Systemic Lupus Erythematosus in T Cell Signaling Pathway | 2.78 | 0.01 | -1.34 | FAS,IL10,IL2,IL6,LEP |
| Role of Hypercytokinemia/hyperchemokinemia in the Pathogenesis of Influenza | 8.37 | 0.07 | -1.63 | CCL2,IFNL2,IL10,IL33,IL5,IL6 |
| Neuroinflammation Signaling Pathway | 4.09 | 0.02 | -2.24 | CCL2,FAS,IL10,IL4,IL6 |

Significant pathways were identified based on 24 differentially expressed cytokines with an absolute Z-Score of ≥ 1 between the healthiest time point and the preceding 8 samples. A z-score = NaN indicates pathways that are constructed in such a way that no activity prediction can currently be made.

**Table S4.** Differentially Expressed Cytokines at the Healthiest Time Point Linked to Canonical Signaling Pathways in Pathogen-Induced Cytokine Storm

| Symbol | Entrez Gene Name | Gene Symbol | Z score | p-value of overlap | Discovery Rate (q-value) | Expected |
| --- | --- | --- | --- | --- | --- | --- |
| LEP | leptin | LEP | 3.619 | 0.0003 | 0.0118 | Up |
| CXCL5 | C-X-C motif chemokine ligand 5 | CXCL5 | 1.367 | 0.172 | 0.97 | Up |
| CXCL1 | C-X-C motif chemokine ligand 1 | CXCL1 | 1.254 | 0.21 | 0.97 | Up |
| IL2 | interleukin 2 | IL2 | 1.192 | 0.233 | 0.97 | Up |
| CCL17 | C-C motif chemokine ligand 17 | CCL17 | 1.112 | 0.266 | 0.97 | Up |
| CCL21 | C-C motif chemokine ligand 21 | CCL21 | 1.043 | 0.297 | 0.97 | Up |
| IL33 | interleukin 33 | IL33 | 0.996 | 0.319 | 0.97 | Up |
| IL4 | interleukin 4 | IL4 | -1.119 | 0.263 | 0.97 | Up |
| IL10 | interleukin 10 | IL10 | -1.179 | 0.238 | 0.97 | Up |
| CCL13 | C-C motif chemokine ligand 13 | CCL13 | -1.201 | 0.23 | 0.97 | Up |
| IL6 | interleukin 6 | IL6 | -1.287 | 0.198 | 0.97 | Up |
| IL9 | interleukin 9 | IL9 | -1.511 | 0.131 | 0.805 | Up |
| CCL27 | C-C motif chemokine ligand 27 | CCL27 | -1.72 | 0.0855 | 0.57 | Up |
| VEGFA | vascular endothelial growth factor A | VEGF | -1.793 | 0.0729 | 0.53 | Up |
| CCL15 | C-C motif chemokine ligand 15 | CCL15 | -2.06 | 0.0394 | 0.35 | Up |
| IL5 | interleukin 5 | IL5 | -2.079 | 0.0376 | 0.35 | Up |
| CXCL9 | C-X-C motif chemokine ligand 9 | MIG | -2.108 | 0.0351 | 0.35 | Up |
| CCL2 | C-C motif chemokine ligand 2 | CCL2 | -2.268 | 0.0233 | 0.35 | Up |
| CCL11 | C-C motif chemokine ligand 11 | CCL11 | -2.752 | 0.006 | 0.119 | Up |

Significant pathways were identified based on 24 differentially expressed cytokines with an absolute Z-Score of ≥ 1 between the healthiest time point and the preceding 8 samples.


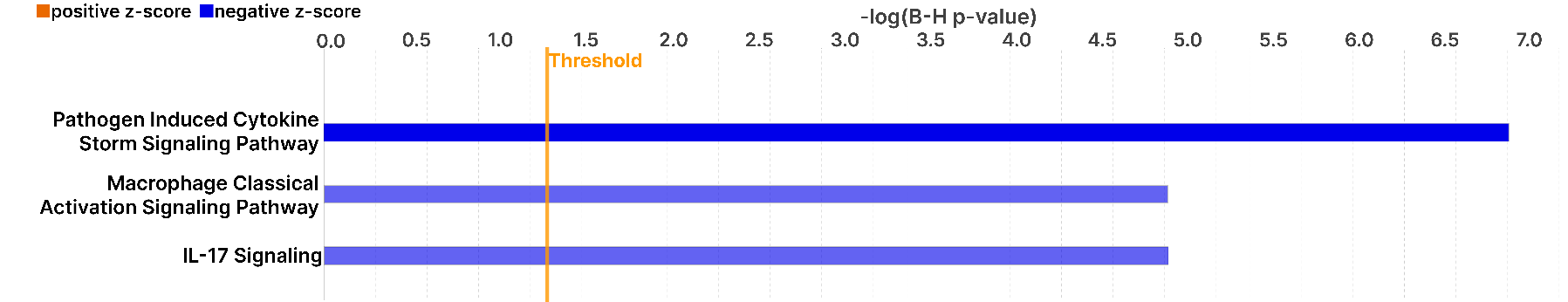


**Figure S2.** IPA canonical pathway analysis of differentially expressed cytokines with an absolute Z-Score of ≥1 and p-value < 0.05 between the healthiest time point and the preceding 8 samples.

**Table S5.** Top Predicted Activated or Inhibited Upstream Regulators Identified by IPA.

| **Upstream Regulator** | **Expr Log Ratio** | **Molecule Type** | **Predicted Activation State** | **Activation z-score** | **p-value of overlap** | **Target Molecules in Dataset** | **Mechanistic Network** |
| --- | --- | --- | --- | --- | --- | --- | --- |
| BCL6 |  | Transcription regulator | Activated | 2.067 | 5.97E-11 | CCL2,FAS,FGF2,IL10,IL4,IL5,IL6,IL9 | 22 (19) |
| IFN Beta |  | Group | Inhibited | -2.393 | 2.11E-07 | CCL2,CXCL1,CXCL5,IL10,IL6,IL9 | 8 (3) |
| PRKCD |  | Kinase | Inhibited | -2.132 | 0.00000116 | CCL2,IL10,IL4,IL6,VEGFA | 20 (21) |
| TLR4 |  | Transmembrane receptor | Inhibited | -2.175 | 5.18E-09 | CCL2,FAS,IL10,IL2,IL4,IL5,IL6,MIF | 18 (13) |
| PLA2G10 |  | Enzyme | Inhibited | -2.425 | 5.22E-12 | CCL2,FAS,IL10,IL4,IL5,IL6,VEGFA | 23 (15) |
| TWIST1 |  | Transcription regulator | Inhibited | -2.211 | 0.00000202 | CCL2,FAS,IL10,IL6,VEGFA | 5 (2) |
| CEBPB |  | Transcription regulator | Inhibited | -2.08 | 2.37E-10 | CCL2,CXCL5,FAS,HGF,IL10,IL2,IL4,IL5,IL6,LEP | 21 (19) |
| IL2 | 1.192 | Cytokine | Inhibited | -2.442 | 2.51E-12 | CCL11,CCL17,CCL2,FAS,FGF2,IL10,IL2,IL4,IL5,IL6,IL9,VEGFA | 21 (16) |
| FCGR2A |  | Transmembrane receptor | Inhibited | -2.215 | 2.3E-09 | CCL2,FAS,IL10,IL6,VEGFA | 20 (23) |
| CYBB |  | Enzyme | Inhibited | -2.207 | 6.59E-10 | CCL2,IL2,IL4,IL5,IL6 | 20 (22) |
| GATA3 |  | Transcription regulator | Inhibited | -2.393 | 3.75E-09 | IL10,IL2,IL4,IL5,IL6,IL9,VEGFA | 18 (14) |
| NMU |  | Other | Inhibited | -2.407 | 2.31E-13 | IL10,IL4,IL5,IL6,IL9,LEP | 7 (3) |
| IL18 | 0.793 | Cytokine | Inhibited | -2.176 | 2.65E-17 | CCL11,CCL2,CXCL9,FAS,IL10,IL2,IL4,IL5,IL6,IL9,VEGFA | 22 (18) |
| IL9 | -1.511 | Cytokine | Inhibited | -2.412 | 2.92E-11 | CCL11,CCL2,IL4,IL5,IL9,VEGFA |  |
| MAPK3 |  | Kinase | Inhibited | -2.2 | 7.89E-10 | IL10,IL2,IL4,IL5,IL6,VEGFA | 17 (14) |
| MAP2K3 |  | Kinase | Inhibited | -2.176 | 7.76E-09 | CXCL9,IL10,IL4,IL6,VEGFA | 17 (15) |
| KITLG | 0.138 | Growth factor | Inhibited | -2.209 | 1.85E-07 | CCL2,IL10,IL4,IL5,IL6,IL9 | 20 (20) |
| FOS |  | Transcription regulator | Inhibited | -2.2 | 0.0000222 | CCL2,FGF2,IL10,IL2,IL6,VEGFA | 21 (17) |
| CTNNB1 |  | Transcription regulator | Inhibited | -2.353 | 4.61E-07 | CCL2,CXCL1,FAS,IL10,IL2,IL4,IL6,IL9,VEGFA | 20 (21) |
| ESR1 |  | ligand-dependent nuclear receptor | Inhibited | -2.183 | 0.00000613 | CCL15,CCL2,CXCL1,FAS,IL2,IL5,IL6,LEP,VEGFA | 20 (16) |
| NfkB-RelA |  | Complex | Inhibited | -2 | 1.96E-15 | CCL11,CCL13,CCL2,CXCL9,IL10,IL2,IL6 |  |
| EPO |  | Cytokine |  | 1.981 | 0.0000129 | FAS,FGF2,IL2,IL6,VEGFA | 21 (20) |
| SCGB1A1 |  | Cytokine |  | 1.98 | 1.51E-08 | CCL11,IL10,IL4,IL5 |  |
| etanercept |  | biologic drug |  | 1.969 | 6.67E-08 | CCL2,CCL27,CXCL9,IL6 |  |
| IL11 |  | Cytokine |  | 1.961 | 0.00000102 | IL10,IL4,IL5,IL6 | 18 (18) |
| E2F1 |  | Transcription regulator |  | 1.951 | 0.000156 | FAS,FGF2,LEP,MIF,VEGFA | 8 (3) |
| KLF2 |  | Transcription regulator |  | 1.946 | 0.000034 | CCL2,IL2,IL4,IL6 | 19 (7) |
| SIRT3 |  | Enzyme |  | 1.945 | 9.15E-07 | FAS,IL10,IL6,VEGFA | 19 (12) |
| Ciap |  | Group |  | 1.941 | 7.84E-09 | CCL11,IL10,IL6,IL9 |  |
| HBB |  | transporter |  | 1.89 | 1.68E-18 | CCL11,CCL2,CXCL1,CXCL5,IL10,IL2,IL4,IL5,IL6,IL9 | 19 (7) |
| STAT1 |  | Transcription regulator |  | -1.838 | 2.37E-15 | CCL2,CXCL9,FAS,FGF2,IFNL2,IL10,IL2,IL33,IL4,IL5,IL6,VEGFA | 21 (16) |
| IL23 |  | Complex |  | -1.841 | 1.15E-09 | IL10,IL2,IL4,IL6,IL9 | 11 (8) |
| GLI1 |  | Transcription regulator |  | -1.845 | 0.000193 | FAS,IL10,IL33,IL6,VEGFA |  |
| ERN1 |  | Kinase |  | -1.854 | 0.0000165 | FAS,IL4,IL6,VEGFA | 13 (7) |
| CCL11 | -2.752 | Cytokine |  | -1.86 | 5.75E-21 | CCL11,CCL2,CCL27,CXCL5,CXCL9,FAS,IL4,IL6,IL9,LEP |  |
| CD2 |  | Transmembrane receptor |  | -1.861 | 2.94E-12 | CCL11,FAS,IL10,IL2,IL4,IL5 | 19 (18) |
| CYP19A1 |  | Enzyme |  | -1.886 | 0.00000448 | FGF2,IL6,LEP,VEGFA | 7 (3) |
| CHUK |  | Kinase |  | -1.891 | 4.76E-15 | CCL11,CCL2,CCL21,FAS,IL10,IL2,IL4,IL5,IL6,VEGFA | 21 (15) |
| S100A8 |  | Other |  | -1.914 | 4.36E-10 | CCL11,CCL2,CXCL9,FAS,IL10,IL33,IL6 |  |
| IFIH1 |  | Enzyme |  | -1.914 | 1.96E-07 | CCL11,CCL2,FAS,IL6 |  |
| FCER1G |  | Transmembrane receptor |  | -1.934 | 4E-10 | IL10,IL2,IL4,IL5,IL6 | 16 (15) |
| IFNA2 | -0.269 | Cytokine |  | -1.935 | 2.17E-14 | CCL2,CXCL9,FAS,FGF2,IL10,IL2,IL4,IL5,IL6,VEGFA | 22 (21) |
| MAPKAPK2 |  | Kinase |  | -1.937 | 1.39E-13 | CCL11,CCL2,IL10,IL2,IL4,IL5,IL6 | 15 (12) |
| FOXO1 |  | Transcription regulator |  | -1.938 | 0.00132 | FAS,IL4,IL6,LEP | 19 (14) |
| KLF13 |  | Transcription regulator |  | -1.941 | 2.87E-09 | IL10,IL4,IL5,IL9 |  |
| IKZF1 |  | Transcription regulator |  | -1.944 | 0.000193 | IL10,IL4,IL5,IL6 | 20 (15) |
| IFNGR1 |  | Transmembrane receptor |  | -1.945 | 0.0000252 | CCL17,FAS,IL2,IL6 |  |
| NOD2 |  | Other |  | -1.949 | 9.15E-07 | CCL2,IL10,IL2,IL6 | 20 (22) |
| Aldose Reductase |  | Group |  | -1.951 | 4.43E-09 | IL10,IL4,IL5,IL6 | 11 (4) |
| TNFSF13B |  | Cytokine |  | -1.951 | 0.0000134 | CCL2,FAS,IL10,LEP | 13 (7) |
| CCN1 |  | Other |  | -1.952 | 0.00000165 | CCL2,IL6,MIF,VEGFA | 8 (3) |
| TIGIT |  | Other |  | -1.953 | 5.32E-10 | IL10,IL4,IL5,IL9 | 8 (3) |
| PAEP |  | Other |  | -1.953 | 2.87E-09 | CXCL1,CXCL5,IL2,VEGFA |  |
| MYDGF |  | Cytokine |  | -1.954 | 2.46E-10 | IL10,IL4,IL5,IL9 |  |
| ICOSLG/LOC102723996 |  | Other |  | -1.955 | 1.29E-08 | IL10,IL4,IL5,IL6 | 10 (3) |
| BTK |  | Kinase |  | -1.958 | 1.05E-07 | CXCL9,IFNL2,IL10,IL5,IL6 |  |
| CCL4 | -0.312 | Cytokine |  | -1.96 | 5.41E-09 | CCL2,IL4,IL5,IL6 | 20 (13) |
| STIM1 |  | ion channel |  | -1.964 | 6.56E-07 | IL10,IL6,TPO,VEGFA |  |
| PLP1 |  | Other |  | -1.969 | 2.66E-10 | CCL2,IL10,IL2,IL4,IL5 | 14 (5) |
| IRF8 |  | Transcription regulator |  | -1.969 | 0.00000132 | CXCL9,FAS,IL4,IL5,IL6 | 20 (13) |
| TP63 |  | Transcription regulator |  | -1.976 | 0.000339 | FAS,HGF,IL6,TPO,VEGFA |  |
| TICAM1 |  | Other |  | -1.978 | 3.13E-07 | CCL2,FAS,IL10,IL4,IL6 | 20 (18) |
| TRAF4 |  | Other |  | -1.982 | 7.27E-11 | CCL11,IL4,IL5,IL6,IL9 |  |
| ADRB2 |  | G-protein coupled receptor |  | -1.982 | 2.47E-07 | IL10,IL2,IL6,LEP | 14 (14) |
| NPY |  | Other |  | -1.982 | 4.86E-07 | FAS,IL10,IL6,LEP | 20 (15) |
| GATA2 |  | Transcription regulator |  | -1.982 | 0.00000335 | CCL2,CCL21,IL4,IL5,IL6,VEGFA | 14 (7) |
| Akt |  | Group |  | -1.987 | 4.15E-09 | CCL2,FAS,IL10,IL4,IL5,IL6,LEP,VEGFA | 16 (15) |

Upstream Regulators analysis was conducted based on 24 differentially expressed cytokines with an absolute Z-Score of ≥1 during health improvement from Extremely Severe D to A. Positive z-scores predict activation, while negative z-scores indicate inhibition. The 'Target Molecules' column represents differentially expressed cytokines identified in our longitudinal cytokine analyses. The p-value of overlap is extracted from the IPA algorithm.

**
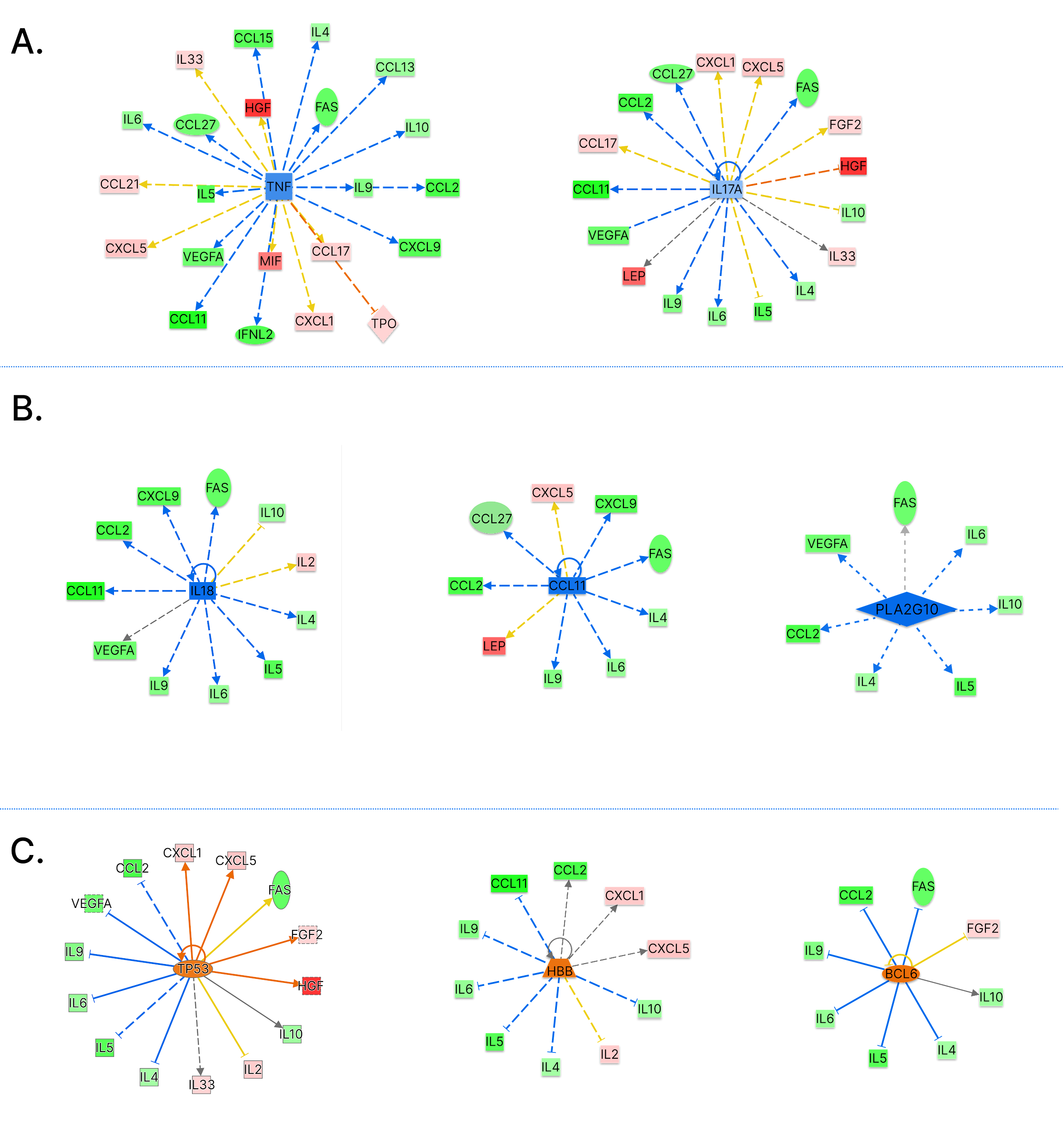
**

**Figure S3: Ingenuity Pathway Analysis (IPA).**  **A)** IPA canonical pathway analyses revealed Predicted inhibition of TNF and IL17A based on differentially expressed cytokines at the healthiest time point to the average of less healthy time points. The depicted pathways passed the B-H multiple testing correction (p-value<0.05) and an absolute IPA z-score greater than 0.8. The color-coded bars indicate the predicted activation (orange) or inhibition (blue) of each pathway. **B)** Upstream Regulator Analysis - Inhibition: This panel shows the IPA upstream regulator analysis predicting the inhibition of IL18, CCL11and PLA2G10 signaling pathways. **C)** Upstream Regulator Analysis - Activation: This panel displays the IPA upstream regulator analysis predicting the activation of BCL6, HPP, and TP53 signaling pathways.

**Table S6.** Differently expressed cytokines associated with CCL11 upstream regulator

| Target | Expr Log Ratio | Molecule Type | CCL11 |
| --- | --- | --- | --- |
| CCL2 | -2.268 | cytokine | Inhibited |
| CCL27 | -1.72 | other | Inhibited |
| CXCL5 | 1.367 | cytokine | Activated |
| CXCL9 | -2.108 | cytokine | Inhibited |
| FAS | -1.91 | transmembrane receptor | Inhibited |
| IL4 | -1.119 | cytokine | Inhibited |
| IL6 | -1.287 | cytokine | Inhibited |
| IL9 | -1.511 | cytokine | Inhibited |
| LEP | 3.619 | growth factor | Activated |

Nine of 24 differentially expressed cytokines during health improvement from extremely severe D to A interact with the CCL11 signaling pathway, as identified by IPA Upstream Regulator Analysis. Predicted inhibition of CCL11 signaling during this transition is based on cytokine expression and the p-value for overlap.


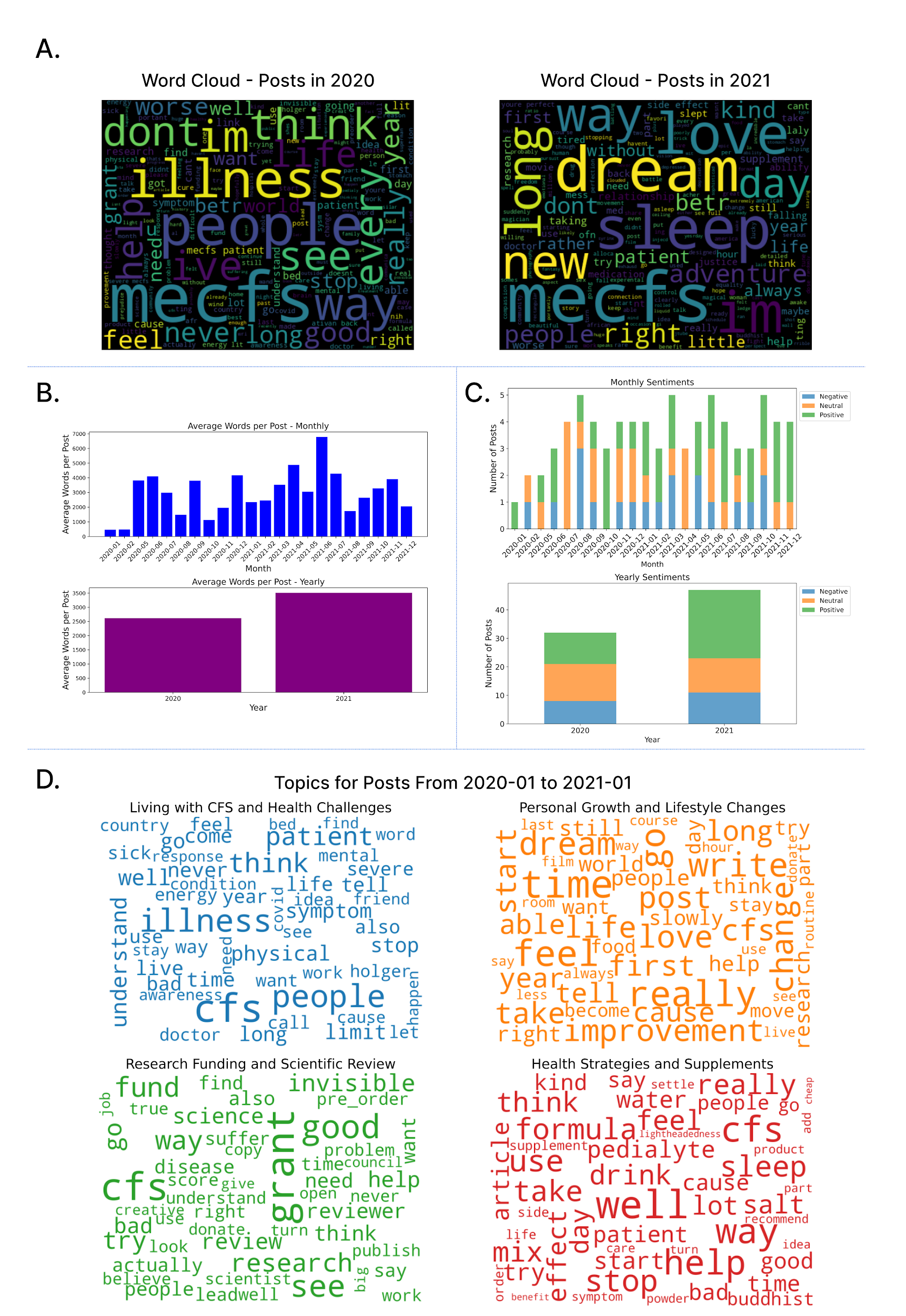


**Figure S4. Topic and Sentiment Analysis of Patients Blog Posts.** **A)** Word clouds illustrating the most frequently used words in blog posts from the years 2020 and 2021, providing a visual snapshot of prevalent themes and topics during this period. **B)** The average length of posts written monthly and yearly from 2020 to 2021, used as an indicator of social and media activity. **C)** Sentiments of blog posts made in 2020 and 2021, number of posts are separated based on overall sentiment, positive (green), neutral (orange) and negative (blue). The top figure shows sentiments of posts made per month and the bottom figure shows sentiments of posts made per year. **D)** Topic analysis reveals four main themes: challenges with ME/CFS, personal experiences, research discussions, and health management strategies, represented by word clouds highlighting significant term

**Table S7**. **Contingency Table and Fisher Exact Test Results for Sentiment Distribution in 2020 and 2021**

| Contingency Table | | | |
| --- | --- | --- | --- |
| **Year \ Sentiment** | **Negative** | **Neutral** | **Positive** |
| **2020** | 8 | 13 | 11 |
| **2021** | 11 | 13 | 2 |
|  |  |  |  |
| **Fishers Exact Test** | | | |
|  | | **Odds Ratio** | **p-Value** |
| **Negative vs. Neutral** | | 0.7273 | 0.7636 |
| **Negative vs. Positive** | | 0.1322 | 0.0278 |
| **Neutral vs. Positive** | | 0.1819 | 0.0449 |

The Fisher exact test was employed to quantify differences in sentiments between 2020 and 2021, providing statistical insights. The results show significant distinctions in sentiment distribution. In 2021, the Negative vs. Positive sentiment comparison exhibits a markedly lower odds ratio, signifying a substantial decrease in Negative sentiments compared to Positive ones. Similarly, the comparison between Neutral and Positive sentiments indicates a noteworthy reduction in Neutral sentiments in 2021 relative to Positive sentiments, indicating a significant shift in sentiment expression over time.

| **ChatGPT 3.5** |
| --- |
|  |
| Prompt: |
| What come to your mind if I tell you these are the most used words in someone’s blog:  CFS, people, illness, think, stop, worse, never, help, long, even, feel, severe, will, make, one, going, way, much, patients, research, think, thing, really. |
|  |
| Answer: |
| Based on these most used words, it seems like the person’s blog may revolve around Chronic Fatigue Syndrome (CFS) and its impact on people’s lives. The words suggest a focus on the challenges and struggles associated with the illness, including feelings of severity, the search for help, and the impact on daily life.  The repetition of words like “think”, “feel” and “way” may indicate a reflective and introspective tone in the blog. Additionally, the inclusion of “patients” and “research” suggests an interest in sharing information and experiences related to CFS, possibly to contribute to ongoing research or provide support to others facing similar challenges. |

**Figure S5**: **Integration of Word Cloud and ChatGPT for Efficient Analysis of Personal Narratives in An Extremely ME/CFS Patient Blog Posts.** The 23 most frequently used words, extracted from the patient's blog posts' word cloud, were utilized as input for ChatGPT to discern the patient's main message. The primary objective is to improve communication between ME/CFS patients and medical and research teams, especially for those facing verbal communication challenges due to disease severity. ChatGPT analysis emphasized a predominant focus on the challenges, severity, and profound impact of ME/CFS on individuals' lives. Additionally, the patient shows a strong interest in disseminating information and sharing personal experiences related to ME/CFS, contributing to ongoing research efforts. This approach serves as a valuable tool for monitoring health transitions, especially in non-verbally communicative patients, offering insights into personal well-being, mental health struggles, coping mechanisms, passions, and hobbies. It provides a window into challenges, overcoming obstacles, and strategies for resilience, offering a holistic view that can aid personalized medicine and improve communication between patients, medical teams, and researchers

| **ChatGPT 3.5** |
| --- |
|  |
| Prompt 1: |
| Based on these words ordered by frequency from a word cloud analysis on blog posts made in 2020 for a patient, what overall feeling would you get on the patient's state of health? ['mecfs', 'people', 'illness', 'way', 'im', 'think', 'see', 'dont', 'ive', 'every', 'life', 'help', 'good', 'never', 'really', 'year', 'feel', 'long', 'worse', 'stop', 'grant', 'betr', 'want', 'world', 'well', 'u', 'need', 'right', 'mecfs patient', 'research', 'lot', 'going', 'symptom', 'use', 'understand', 'cant', 'day', 'able', 'go', 'idea', 'cause', 'thought', 'patient', 'lit', 'got', 'bed', 'first', 'link', 'find', 'physical', 'doctor', 'come', 'sick', 'made', 'afr', 'work', 'ting', 'country', 'invisible', 'energy lit', 'energy', 'still', 'actually', 'keep', 'portant', 'person', 'nih', 'little', 'cure', 'called', 'post', 'mental', 'last', 'living', 'always', 'start', 'awareness', 'back', 'friend', 'take', 'may', 'didnt', 'holger', 'word', 'condition', 'provement', 'full', 'real', 'mind', 'trying', 'new', 'let', 'youre', 'sysm', 'one', 'le', 'change', 'ativan', 'doesnt', 'covid', 'please', 'brain', 'reason', 'month', 'case', 'stay', 'product', 'care', 'part', 'funding', 'wind', 'health', 'try', 'best', 'home', 'preorder', 'fund', 'nt', 'severe mecfs', 'severe', 'without', 'kind', 'whole', 'yet', 'stomach', 'response', 'formula', 'night', 'bad', 'room', 'problem', 'talk', 'slowly', 'wand', 'difficult', 'past', 'hospital', 'extremely', 'already', 'wri', 'silar', 'lead', 'light', 'face', 'enough', 'thats', 'community', 'continue', 'put', 'must', 'al', 'feeling', 'might', 'used', 'felt', 'look', 'outside', 'family', 'superman', 'sure', 'communica', 'huge', 'prejudice', 'shes', 'hopefully', 'situation', 'another', 'science', 'hard', 'creat', 'thinking', 'havent', 'letr', 'recently', 'sta', 'number', 'two', 'happen', 'suffering', 'treatment', 'healthy', 'maybe', 'history', 'progress', 'possible', 'experienced', 'happening', 'public', 'whaver', 'course', 'fact', 'pedialy', 'turn', 'isnt', 'order', 'article', 'probably', 'ofn', 'book', 'great'] |
|  |
| Answer 1: |
| Based on the words ordered by frequency from the word cloud analysis, the overall feeling about the patient's state of health in 2020 appears to be challenging and difficult. The prominent terms such as "mecfs" (indicating a reference to Myalgic Encephalomyelitis/Chronic Fatigue Syndrome), "illness," "worse," "stop," "symptom," "sick," and "severe mecfs" suggest a patient grappling with health issues, including symptoms that may be severe. There is a notable emphasis on challenges and difficulties related to the patient's health, as well as expressions of seeking help, understanding, and coping with the condition. Overall, the word cloud suggests a complex and demanding health situation for the patient in 2020. |
|  |
| Prompt 2: |
| Based on these words ordered by frequency from a word cloud analysis on blog posts made in 2021 for a patient, what overall feeling would you get on the patient's state of health? ['mecfs', 'life', 'dont', 'people', 'feel', 'year', 'way', 'world', 'every', 'u', 'im', 'help', 'day', 'name', 'mecfs patient', 'need', 'think', 'use', 'illness', 'new', 'love', 'go', 'betr', 'take', 'able', 'cant', 'still', 'keep', 'back', 'come', 'right', 'experience', 'put', 'going', 'patient', 'al', 'post', 'long', 'hope', 'feeling', 'want', 'first', 'without', 'afr', 'ive', 'body', 'well', 'really', 'suffering', 'let', 'stomach', 'work', 'find', 'mind', 'person', 'try', 'ting', 'possible', 'moment', 'never', 'little', 'worse', 'used', 'sick', 'binder', 'portant', 'part', 'best', 'sound', 'severe mecfs', 'le', 'change', 'research', 'good', 'always', 'kind', 'mean', 'happen', 'room', 'sta', 'point', 'got', 'made', 'lot', 'ofn', 'actually', 'death', 'around', 'toher', 'drug', 'whole', 'great', 'please', 'stop', 'away', 'bed', 'didnt', 'doesnt', 'routine', 'continue', 'health', 'app', 'button', 'loss', 'human', 'look', 'hard', 'set', 'left', 'friend', 'different', 'huge', 'l', 'likely', 'funding', 'cause', 'wri', 'relationship', 'understand', 'remember', 'energy', 'lit', 'number', 'letr', 'enough', 'taking', 'thought', 'two', 'felt', 'communica', 'outside', 'crash', 'president biden', 'believe', 'took', 'see', 'caregr', 'easy', 'become', 'sleep', 'die', 'using', 'remo', 'making', 'word', 'thinking', 'rest', 'living', 'dream', 'called', 'crashing', 'cure', 'trying', 'family', 'example', 'whaver', 'link', 'pa', 'though', 'youre', 'constantly', 'doctor', 'whi noise', 'happy', 'support', 'writn', 'incredible', 'yet', 'complely', 'beautiful', 'loved', 'g', 'bit', 'bad', 'open', 'nih', 'affect', 'sense', 'push', 'idea', 'either', 'thats', 'anymore', 'maybe', 'etc', 'crea', 'nt', 'earmuff', 'understanding', 'lost', 'care', 'real', 'last', 'start', 'heart', 'brain', 'difficult', 'somes', 'needed', 'sply'] |
|  |
| Answer 2: |
| Based on the words ordered by frequency from the word cloud analysis of blog posts made in 2021, the overall feeling regarding the patient's state of health appears to be a mix of challenges, hope, and personal reflections. Positive terms such as "love," "hope," "good," "best," and "beautiful" suggest a focus on positive aspects and a sense of resilience. Additionally, the use of words like "try," "possible," and "moment" indicates an ongoing effort to overcome challenges.  On the other hand, terms like "suffering," "hard," "difficult," and "crash" still convey the presence of health struggles. The mention of "severe mecfs," "stomach," and "sick" implies ongoing health issues, while expressions like "keep," "still," and "cant" indicate persistence in facing difficulties.  Overall, the word cloud suggests a nuanced and complex narrative, where the patient is navigating health challenges while maintaining a hopeful and determined outlook. |
|  |
| Prompt 3: |
| How does the 2021 word cloud analysis of blog posts made by the patient in 2021 compare to the 2020 word cloud analysis of the blog posts? |
|  |
| Answer 3: |
| Comparing the 2021 and 2020 word cloud analyses of the patient's blog posts reveals some notable shifts in the patient's focus and emotional tone.  **2021 Word Cloud:**   - Positive terms such as "love," "hope," "good," and "beautiful" suggest a more optimistic and resilient outlook. - Words like "try," "possible," and "moment" indicate an emphasis on efforts to overcome challenges. - While there are still mentions of health struggles ("suffering," "hard," "difficult," and "crash"), there seems to be a more balanced mix of positive and challenging expressions.   **2020 Word Cloud:**   - The dominant terms include "mecfs," "illness," "worse," "stop," and "symptom," highlighting a focus on the difficulties and challenges of the health condition. - Terms like "severe mecfs," "stomach," and "sick" underscore the severity of health issues. - The overall tone appears to be more centered on the hardships of dealing with health problems, with a greater emphasis on struggles.   **Comparison:**   - The 2021 word cloud suggests a shift toward a more positive and hopeful narrative compared to the 2020 word cloud, where there was a more predominant focus on challenges and difficulties. - Expressions of love, hope, and positive outlook in 2021 may indicate a different mindset or a shift in the patient's emotional state. - The presence of terms like "try," "possible," and "moment" in 2021 implies a focus on efforts and potential progress, while the 2020 word cloud emphasized the severity of symptoms and challenges faced.   In summary, the 2021 word cloud analysis suggests a potentially more optimistic and resilient perspective compared to the 2020 analysis, where challenges and hardships took center stage. |

**Figure S6: Longitudinal Word Cloud-ChatGPT Integration for Comparative Analysis of ME/CFS Patient Narratives in 2020 and 2021.** The most frequent words from patient blog posts in both years were utilized as ChatGPT input for longitudinal pattern analysis. ChatGPT identified a shift in emotional state, noting a more positive outlook in 2021, despite the continued presence of some challenging emotions and experiences.


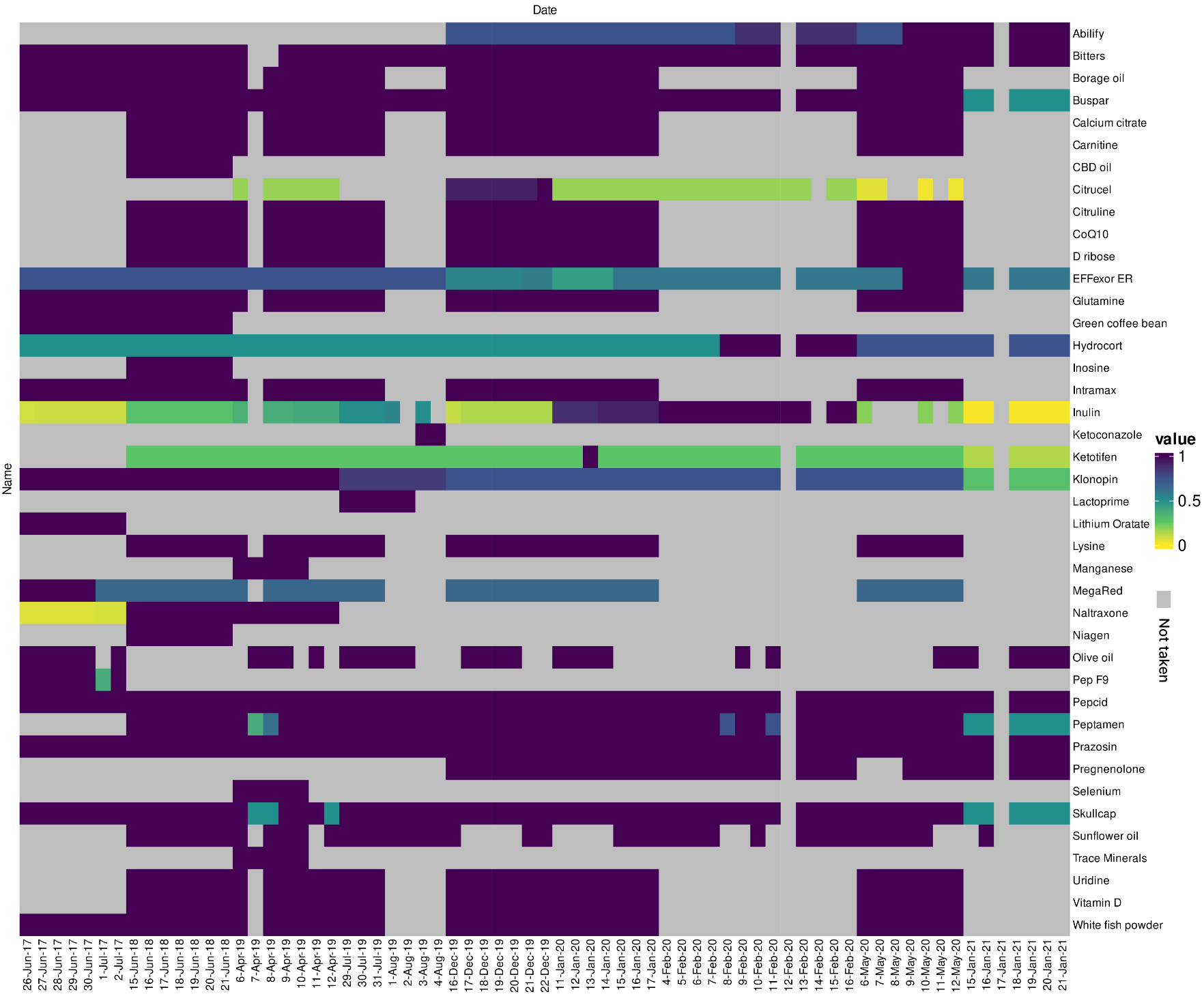


**Figure S7**. Medication usage over time is visualized for all timepoints for the past 4 years of tracking the patient. To facilitate comparison, the dosage of each medication has been standardized over time using min-max scaling. This standardization is represented in a heatmap, gray squares represent no medication dosage recorded.

**Table S8. Pearson Correlation Analysis of Medication Use and Health Improvement from Extremely Severe D to Extremely Severe A.**

| Name | Correlation | P-Value |
| --- | --- | --- |
| Skullcap | -1 | 0 |
| Buspar | -1 | 0 |
| Klonopin | -0.8809 | 0.0204 |
| Borage oil | -0.6325 | 0.1778 |
| Intramax | -0.6325 | 0.1778 |
| Glutamine | -0.6325 | 0.1778 |
| White fish powder | -0.6325 | 0.1778 |
| MegaRed | -0.5998 | 0.2082 |
| Abilify | 0.4825 | 0.3324 |
| D ribose | -0.4472 | 0.3739 |
| Uridine | -0.4472 | 0.3739 |
| Vitamin D | -0.4472 | 0.3739 |
| Citruline | -0.4472 | 0.3739 |
| Calcium citrate | -0.4472 | 0.3739 |
| Lysine | -0.4472 | 0.3739 |
| CoQ10 | -0.4472 | 0.3739 |
| Carnitine | -0.4472 | 0.3739 |
| Inulin | -0.4431 | 0.3789 |
| Olive oil | 0.3162 | 0.5415 |
| Sunflower oil | -0.3162 | 0.5415 |
| Green coffee bean | -0.3162 | 0.5415 |
| Pregnenolone | 0.3162 | 0.5415 |
| Ketotifen | -0.2944 | 0.5711 |
| Peptamen | -0.2928 | 0.5733 |
| Hydrocort | 0.2928 | 0.5734 |
| Citrucel | -0.2895 | 0.5779 |
| Naltraxone | -0.2131 | 0.6852 |
| Pep F9 | -0.2 | 0.704 |
| CBD oil | -0.2 | 0.704 |
| Inosine | -0.2 | 0.704 |
| Lithium Oratate | -0.2 | 0.704 |
| Niagen | -0.2 | 0.704 |
| EFFexor ER | -0.0698 | 0.8954 |
| Selenium |  |  |
| Lactoprime | |  |
| Pepcid |  |  |
| Prazosin |  |  |
| Trace Minerals | |  |
| Manganese | |  |

The comparison of medication doses on the day of blood draw showed a positive correlation with a reduction in intake for Skullcap, Buspar, and Klonopin, as well as an increase in low-dose Abilify and health improvement. Abilify correlation with health improvement did not reach standard significance.
